# A Transferable Force Field for Predicting Adsorption and Diffusion of Water in Cationic Zeolites with Coupled Cluster Accuracy

**DOI:** 10.1021/acsphyschemau.5c00038

**Published:** 2025-08-08

**Authors:** Salah Eddine Boulfelfel, Hanjun Fang, Alan S. S. Daou, Peter I. Ravikovitch, David S. Sholl

**Affiliations:** † School of Chemical and Biomolecular Engineering, 1372 Georgia Institute of Technology, Atlanta, Georgia 30332-0100, United States; ‡ 428028 ExxonMobil Technology and Engineering Company, 1545 Route 22 East, Annandale, New Jersey 08801, United States; § 6146 Oak Ridge National Laboratory, Oak Ridge, Tennessee 37830, United States

**Keywords:** zeolite, water, adsorption, diffusion, force field

## Abstract

We present a transferable force field for water in proton-exchanged, alkali (Li, Na, K, Rb, and Cs) metal-exchanged, and alkaline-earth (Mg, Ca, Sr, and Ba) metal-exchanged zeolites. The fitting methodology is based on adsorbate–adsorbent interaction energies obtained from periodic density functional theory calculations and corrected using the coupled-cluster method applied to small model clusters. To ensure an accurate prediction of both adsorption and diffusion properties of water, sets of configurations that sample both adsorption sites and intracrystalline hopping transition states were used in the fitting. The quality of the force field is assessed for a wide range of zeolites with different topologies and chemical compositions, demonstrating good agreement between theoretical predictions and experimental measurements of water adsorption and diffusion.

## Graphical abstract

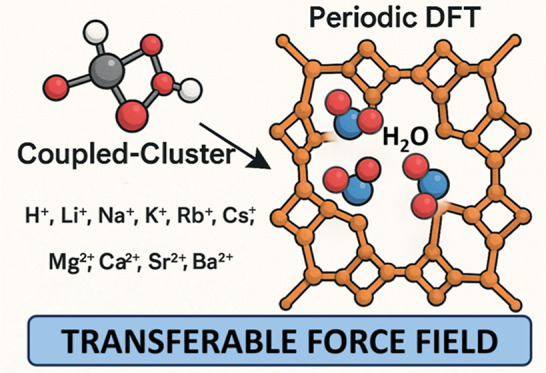

## 1 Introduction

Zeolites are microporous crystalline materials with a wide range of applications as catalysts, ion exchangers, molecular sieves, and detergents.^1,2^ Pure silica zeolites are hydrophobic but both natural and synthetic aluminosilicates are highly hydrophilic due to the presence of cations.^3^ Structural stability of zeolites can be affected under prolonged exposure to water especially in the presence of acidic gases such as carbon dioxide and hydrogen sulfide.^4,5^ The elimination of water prior to industrial operations is in particular very important for the oil and gas sector, where water vapor is considered as a major contaminant in raw natural gas. Even in small quantities, water may cause transportation and processing issues such as gas hydrate build-up, leading to equipment and pipeline blockage, lowering of natural gas heating value, and corrosion.^4,6^

Water removal can be carried out using adsorption processes by temperature and/or pressure swing.^6^ Due to strong water–zeolite interactions, high regeneration temperatures (≥350 °C) are usually required to ensure a thorough regeneration of conventional zeolites such as 4A and 13X. At high temperatures, the mixture of humidity and some adsorbates like heavy hydrocarbons during regeneration scheme can lead to a slow and irreversible breakdown of the crystal structure and adsorbent deactivation.^4^ Some zeolites like CsNa-RHO and KNa-LTA showed potential for deep dehydration with low regeneration temperature without a significant compromise of adsorption capacity.^5,7,8^ The identification of zeolites for dehydration requires a fundamental understanding of adsorption and diffusion processes in zeolites of different chemical compositions and topologies.

Using molecular simulations to accurately predict adsorption isotherms, heats of adsorption and self-diffusivities of water in cationic zeolites require force fields capable of reproducing energies from quantum mechanical calculations, such as density-functional theory with coupled-cluster corrections.^9–14^ Offering reliable predictions that require no experimental input is crucial when screening cationic zeolites for separation applications where the distribution of extra-framework cations is experimentally unknown.^11^ To the best of our knowledge, a force field that reliably describes the adsorption and diffusion properties of water in cationic zeolites in any topology, any Si/Al ratio, and any chemical composition is still missing.

In this paper, we extend the DFT/CC-derived force field (CCFF)^13,14^ developed previously for the accurate prediction of adsorption and diffusion of hydrocarbons and small molecules in zeolites to water. The details of the fitting methods and the validation of different components of the force field are discussed in Section 2. Our new force field covers zeolites exchanged with protons and both monovalent (Li, Na, K, Rb, and Cs) and divalent cations (Mg, Ca, Sr, and Ba) in addition to pure silica structures and can be used for zeolites with any topology, any Si/Al ratio, and any cation composition. This transferability was achieved without compromising the accuracy of predictions for the adsorption or diffusion properties of water, as demonstrated for many zeolites in Section 3. CCFF results were compared to the available experimental measurements of adsorption isotherms and heats of water vapor in various zeolites in Section 3.1 and experimental data of water diffusion collected using microscopic techniques such as PFG NMR and QENS in Section 3.2. The effect of some important experimental conditions such as the presence of binder in zeolite samples and the temperature of dehydration used prior to measurements is also discussed.

## 2 Materials and Simulation Methods

We have previously implemented an approach to developing first-principles-based force fields, called CCFF, for molecules in zeolites where fully periodic frameworks were used to represent the adsorbent structures, and QM calculations at the DFT/CC (density functional theory/coupled cluster) level were performed for hundreds or thousands of configurations of adsorbate molecules scattered throughout the frameworks.^10,12–15^ The CCFF accurately predicted experimentally observed adsorption and diffusion properties and showed good transferability across different zeolite topologies. In this paper, we extend the CCFF for H_2_O in monovalent and divalent cation-exchanged zeolites by fitting to DFT/CC data.

As in most classical simulations of adsorption in cation-exchanged zeolites,^16,17^ we assume that the zeolite frameworks are rigid since the influence of framework flexibility is small for adsorption properties of small molecules.^18^ Extra-framework cations, however, are allowed to move. Therefore, four types of pairwise interactions are considered in this work: H_2_O–H_2_O, H_2_O–zeolite, cation–framework, and cation–cation. For H_2_O–H_2_O interactions, the SPC/E model was adopted because it describes the bulk properties of pure water with reasonable accuracy.^19^ Many more complex FFs for water exist, but SPC/E represents a good trade-off between complexity and accuracy for all possible configurations of water molecules. In the following section, we focus on developing force field potentials that account for H_2_O–zeolite, cation–framework, and cation–cation interactions.

### 2.1 Dispersion-Corrected Density Functional Theory Calculations

Periodic DFT calculations were performed using the VASP code^20,21^ based on the projector augmented wave formalism and pseudopotentials.^22,23^ A kinetic energy cutoff of 520 eV was used for the plane-wave basis set to represent the valence electrons of atoms. Because of the large unit cells of the zeolites used in the calculations, only a single *k*-point at the Γ-point of the Brillouin zone was used. The Perdew–Burke–Ernzerhof (PBE) exchange and correlation functional with the D2 dispersion correction from Grimme^24^ was used for adsorbate–adsorbent DFT calculations.

To define interaction energies between molecules and zeolites, the coupled-cluster corrected density functional theory (DFT/CC) method was used.^25^ This method assumes that the interaction can be decomposed as a sum of pairwise interactions between atoms and uses corrections accounting for the difference between coupled cluster results with large basis sets and DFT results for sets of judiciously chosen interacting molecules and clusters representing the zeolite.^26,27^ All coupled-cluster correction curves used in this work for adsorbate–framework interactions were taken from our previous work.^13,14^ We noticed that the DFT/CC correction curves for the atoms of H_2_O with framework Si atoms listed in ref 13’s Supporting Information were incorrect. However, all our calculations in ref 13 used the correct DFT/CC correction curves. We have included these correct values in Table S1 in the Supporting Information. No correction was applied to adsorbate–cation interactions, a reasonable approach given that the van der Waals contribution to these pairwise interactions is small.^28^

### 2.2 Deriving Force Fields from DFT/CC Energies

As in our previous work on H_2_O in siliceous zeolites,^13^ we assume the interactions between each atom in H_2_O and a cation-exchanged zeolite are represented by pairwise van der Waals (vdW) and Coulombic terms

1 $$E_{CCFF}\left(R_{ij}\right)=E_{vdW}+E_{Coul}=s_{12}\frac{C_{12}^{ij}}{R_{ij}^{12}}-s_{6}\frac{C_{6}^{ij}}{R_{ij}^{6}}+\frac{q_{i}q_{j}}{R_{ij}}$$

where *R*_*ij*_ is the distance between the two atoms. *q*_*i*_ and *q*_*j*_ represent the atomic charges. *C*_6_^*ij*^ and *C*_12_^*ij*^ are the attractive and repulsive coefficients with values adopted from work by Grimme’s work (for *C*_6_^*ij*^) or determined by a simple relation (for *C*_12_^*ij*^).^9,10,24^ *s*_12_ and *s*_6_ are global scaling factors that are fitted to allow the closest correspondence between DFT/CC interaction energies and the classical force field in a least-squares sense. This approach neglects polarizability and any influence of many-body contributions. It is of course possible to construct force fields that include these effects, but the approach above has the advantage of simplicity, and we show below that the resulting force field accurately reproduces experimental data in a diverse set of materials.

The density-derived electrostatic and chemical method (DDEC6) was used for the atoms of zeolite framework and extra-framework cations based on DFT electronic densities.^29,30^ This charge assignment method was specifically developed, in part, to reliably reproduce the electrostatic potential inside porous materials. The atomic charges are shown in Table 1. The charges on atoms of water molecule were taken from the work of Berendsen et al.^19^

**Table 1:** Atomic Charges of Atoms of Cation-Exchanged Zeolites and Water Used in CCFF

atom type	*q* (e)	note
Si	1.8708	
Al	1.7906	
O_*z*_^Si^ (–Si–O–Si–)	–0.9354	
O_*z*_^Al^ (−Si–O–Al−)	–1.1427^a^	Na, K, Rb, Cs
O_*z*_^Al^ (−Si–O–Al−)	–1.1288^a^	Li
O_*z*_^Al^ (−Si–O–Al−)	–1.1054^a^	Mg, Ca, Sr, Ba
O_*z*_^Al^ (−Si–O–Al−)	–1.0284^a^	proton
proton	0.4522	
Li	0.8538	
Na	0.9094	
K	0.9094	
Rb	0.9094	
Cs	0.9094	
Mg	1.5204	
Ca	1.5204	
Sr	1.5204	
Ba	1.5204	
O_w_	–0.8476^b^	SPC/E model^19^
H_w_	0.4238^b^	SPC/E model^19^

a Charge on O_*z*_^Al^ (–Si–O–Al–) for zeolites of mixed cations is calculated based on the composition.

b Charges on Ow and Hw were taken from the SPC/E model.^19^

For systems with one type of cation, using the values listed in Table 1 to ensure charge neutrality is straightforward (e.g., 4A zeolite, with chemical formula Na_96_Si_96_Al_96_O_384_, corresponds to 0.9094 × 96 + 1.8708 × 96 + 1.7906 × 96–1.1427 × 384 with *q*[O_*z*_^Al^] = −1.1427 e). For systems with two types of cations or more, the charge on the octahedron on O_*z*_^Al^ (–Si–O–Al–) must be adjusted to maintain charge neutrality (e.g., 5A zeolite, with chemical formula Na_32_Ca_32_Si_96_Al_96_O_384_, corresponds to 0.9094 × 32 + 1.5204 × 32 + 1.8708 × 96 + 1.7906 × 96–1.117833 × 384 with *q*[O_*z*_^Al^] = −1.117833 e).

The vdW terms are expressed using the Lennard-Jones (LJ) 12-6 potential with the form

2 $$E_{LJ}\left(R_{ij}\right)=4\varepsilon_{ij}\left[{\left(\frac{\sigma_{ij}}{R_{ij}}\right)}^{12}-{\left(\frac{\sigma_{ij}}{R_{ij}}\right)}^{6}\right]=\frac{A_{ij}}{R_{ij}^{12}}-\frac{B_{ij}}{R_{ij}^{6}}$$

where ε_*ij*_ and σ_*ij*_ are the energy and size parameters, and *A*_*ij*_ and *B*_*ij*_ are the repulsion and attraction parameters between atom *i* in H_2_O and atom *j* in a zeolite. Once *s*_12_ and *s*_6_ in eq 1 are obtained, the vdW term can be straightforwardly converted to ε_*ij*_ and σ_*ij*_ or *A*_*ij*_ and *B*_*ij*_ in eq 2.

For the vdW term, only the interactions of the oxygen atom of H_2_O with zeolite framework oxygens and extra-framework cations were considered: O_w···O_*z*_ and O_w···cation. The interactions involving Si and Al atoms of the zeolite framework and H atoms of H_2_O were only considered through the effective potential with O_w, O_*z*_, and cation. To ensure the FF parameters are transferable, the scaling factors *s*_12_ and *s*_6_ for O_w···O_*z*_, that were obtained in our earlier work for H_2_O on siliceous zeolites^13^ were retained for cation-exchanged zeolites.

### 2.3 Force Field Fitting Procedure

We used primitive cells of cubic M-LTA structures (Si/Al = 1, M_24_Al_24_Si_24_O_96_ for M = Li, Na, K, Rb, Cs, and proton, and M_12_Al_24_Si_24_O_96_ for M = Mg, Ca, Sr, and Ba) as models for force field development. For monovalent cations, their initial 24 positions are occupied as follows: 16 in 6-ring windows, 6 in 8-ring windows, and 2 positioned in 4-ring windows. For divalent cations, all 12 were in 6-ring windows. The unit cells were fully optimized (size, shape, and atomic positions) by using plane wave DFT at the PBE-D3 level.

CCFF parameters for O_w···cations were determined using an iterative approach. First, 400 H_2_O configurations from GCMC simulations (*T* = 300 K and *P* = 10 kPa) were generated in the zeolite models. For each configuration, the interaction energy was calculated using DFT/CC, where the PBE energy was obtained from VASP and the CC corrections were adopted from our previous work for H_2_O in silica zeolites.^13^ CC corrections for H_2_O with cations were not included.^28^

In the FF fitting, the residual standard deviation (RSD) is minimized

3 $$RSD=\sqrt{\frac{\sum_{k}^{n}{\left(E_{FF}^{k}-E_{DFT/CC}^{k}\right)}^{2}}{n-2}}$$

where *E*_FF_^*k*^ and *E*_DFT/CC_^*k*^ are the energies calculated at the force field and DFT/CC levels, respectively, and *n* denotes the number of configurations. The mean deviation (MD) is also calculated after the parametrization

4 $$MD=\frac{\sum_{k}^{n}\left(E_{FF}^{k}-E_{DFT/CC}^{k}\right)}{n}$$

The RSD and MD can give an overall evaluation of the performance of the fitted FF in reproducing the QM data.

The fitted parameters were then used in the next iteration of GCMC simulations to generate a new set of 400 H_2_O configurations, and the fitting procedure was repeated until the changes of the parameters were less than 3%, the defined convergence criteria. If the convergence is not met after five (or more for a few cases) iterations, the configurations of the last four iterations (a total of 1600) were used to get the final FF parameters. Numerical tests showed that increasing the number of H_2_O configurations in each iteration did not significantly influence the values of the fitted FF parameters.

The FF parameters used to generate the initial training sets for H_2_O with Na and Ca cations were from Clay Force Field (CLAYFF)^31^ with Lorentz–Berthelot mixing rules for cross species interactions. When fitting the first iteration for other cations, O_w···Na and O_w···Ca parameters obtained from the previous iteration were used as the initial parameter set. Tests for several examples indicated that the final fitted parameters are not dependent on the initial parameters used for O_w···cation.

For some systems, especially the divalent cationic zeolites, the fitted values of scaling factor *s*_6_ in eq 1 were negative, which led to unphysical values for σ_*ij*_ in eq 2. This situation arose because of the relatively strong *E*_Coul_ for H_2_O in these zeolites, which in turn requires positive vdW interaction energies from H_2_O···cation to compensate for the total DFT/CC energies during the fitting. In this case, we set *s*_6_ = 0 and only included the repulsion term in fitting for the H_2_O···cation potential.

The resulting FF parameters for H_2_O in cationic zeolites are summarized in Tables 2 and 3. Details of the FF fitting for H_2_O in Na-LTA, K-LTA, Ca-LTA, NaX, and KX with Si/Al = 1 are shown in Figures S1–S5. Overall, the fitting results are reasonably good, with an RSD of 5.5, 4.8, 4.2, 5.1, and 6.1 kJ/mol for H_2_O in Na-LTA, K-LTA, Ca-LTA, NaX, and KX, respectively. For energetically favorable H_2_O configurations in these cationic zeolites, Coulombic energies are the dominant contribution compared to the vdW energies. The distribution of the vdW energy of cation···H_2_O (*E*_vdW cation, to fit_ = *E*_DFT/CC_ – *E*_Coul_ – *E*_vdW SiOAl_) includes more positive values for Ca-LTA compared to those for Na-LTA and K-LTA. This led to negative values of the scaling factor *s*_6_ for O_w···Ca if *s*_6_ was not constrained, as discussed above.

**Table 2:** CCFF Lennard-Jones Parameters for SPC/E Water in Proton, Li, Na, K, and Rb-Zeolites^a^

pairwise interaction	ε/*k*_B_ (K)	σ (Å)
O_w···O_*z*_	41.72	3.385
O_w···Li	204.55	2.288
O_w···Na	101.21	2.727
O_w···K	190.00	3.031
O_w···Rb	60.89	3.334
O_w···Proton	2900.26	1.588

a The parameters for the oxygen atom of water (O_w) with framework oxygen atoms (O_*z*_) were adopted from our previous work.^13^

**Table 3:** CCFF Lennard-Jones Parameters for SPC/E Water in Cs, Mg, Ca, Sr, and Ba-Zeolites^a^

pairwise interaction	*A*/*k*_B_ (K·Å^12^)	*B*/*k*_B_ (K·Å^6^)
O_w···Cs	2.4915 × 10^9^	0
O_w···Mg	1.5227 × 10^7^	0
O_w···Ca	9.2742 × 10^7^	0
O_w···Sr	1.8973 × 10^8^	0
O_w···Ba	5.2127 × 10^8^	0

a The parameters for the oxygen atom of water (O_w) with framework oxygen atoms (O_*z*_) were adopted from our previous work.^13^

### 2.4 Interactions between Cations and Zeolite Framework

For cation–framework interactions, except proton–framework interactions, we used the Buckingham potential plus a Coulombic term in the following form^10,14,32^

5 $$E_{{FF}^{\prime }}\left(R_{ij}\right)=E_{Buck}\left(R_{ij}\right)+E_{Coul}=A_{ij}\exp\left(-B_{ij}R_{ij}\right)-\frac{C_{ij}}{R_{ij}^{6}}+\frac{q_{i}q_{j}}{R_{ij}}$$

where *A*_*ij*_, *B*_*ij*_, and *C*_*ij*_ are the Buckingham parameters between the cation and the framework oxygen atom in a zeolite, and *q*_*i*_ and *q*_*j*_ represent the atomic charges of framework atoms and cations. For proton–framework interactions, we use a Morse potential plus a Coulombic term in the following form

6 $$\begin{array}{l} E_{{FF}^{\prime }}\left(R_{ij}\right)=E_{Morse}\left(R_{ij}\right)+E_{Coul}\\ =D_{ij}\left\{\exp\left[\alpha_{ij}\left(1-\frac{R_{ij}}{r_{0ij}}\right)\right]-2\exp\left[\frac{\alpha_{ij}}{2}\left(1-\frac{R_{ij}}{r_{0ij}}\right)\right]\right\}+\frac{q_{i}q_{j}}{R_{ij}} \end{array}$$

where *D*_*ij*_, α_*ij*_, and *r*_0*ij*_ are the Morse parameters between proton and framework oxygen atoms. In our FF vdW interactions for cation–cation, cation–T (T = Si and Al) were not explicitly considered but were accounted for through the effective potential with framework oxygen atoms. This approximation is reasonable because repulsive Coulombic forces prevent these species from approaching each other.

The Buckingham parameters for monovalent cations (Li, Na, K, Rb, and Cs) and Ca were taken from our previous work.^14,33^ The parameters for divalent cations (Mg, Sr, and Ba) were derived using a similar procedure. 500 random translation moves were performed for one of the cations with a maximum displacement of 1 Å, while holding all other cations and all framework atoms fixed in their equilibrium positions and computed energies using PBE-D2. To avoid net framework charges in fitting, the cation–framework interactions were fitted to relative energies using the framework with all cations at their experimental positions as the reference state. For each configuration, Coulombic energies were computed by using the DDEC6 charges. Relative Coulombic energies were calculated by subtracting the Coulombic energy for the reference state from the Coulombic energy for each configuration. Relative PBE-D2 energies were calculated by the same method for each configuration. Subtracting the relative Coulombic energy from the relative PBE-D2 energy gave the relative vdW energy. Relative vdW energies were fit to a Buckingham potential. Parity plots comparing the fitted energies to PBE-D2 energies for Mg, Sr, and Ba are shown in Figure S6. The fitted Buckingham parameters are summarized in Table 4.

**Table 4:** Buckingham and Morse Parameters for Cation–Framework Interactions^a^

cross species	*A*/*k*_B_ (K)	*B* (Å^–1^)	*C*/*k*_B_ (K·Å^6^)
Li–O_*z*_	3.516 × 10^7^	4.723	1.353 × 10^5^
Na–O_*z*_	5.581 × 10^7^	3.985	9.167 × 10^5^
K–O_*z*_	6.967 × 10^7^	3.475	2.617 × 10^6^
Rb–O_*z*_	4.150 × 10^7^	3.228	2.221 × 10^6^
Cs–O_*z*_	4.420 × 10^7^	2.844	6.499 × 10^6^
Mg–O_*z*_	8.321 × 10^7^	4.200	1.118 × 10^6^
Ca–O_*z*_	4.645 × 10^7^	3.811	4.193 × 10^5^
Sr–O_*z*_	4.697 × 10^7^	3.545	6.341 × 10^5^
Ba–O_*z*_	5.120 × 10^7^	3.291	1.246 × 10^6^
	D/k_B_ (K)	α	*r*_0_ (Å)
proton–O_*z*_	20124.64	6.1041	1.0888

a CCFF parameters for monovalent cations were taken from our previous work.^14^

### 2.5 Parallel Tempering Simulations

Adsorption isotherms in cationic zeolites have been shown to be sensitive to the positions of extra-framework cations.^11,34–36^ Therefore, it is important that the initial cation positions in simulations of this kind are properly equilibrated, especially for zeolites with mixed cations such as 5A (NaCaA). Following the work of Fang et al.,^11^ favorable extra-framework cation positions were determined for our simulations of adsorption isotherms using parallel tempering as implemented in RASPA.^37^ For each system, a set of nine structural replicas were sampled at *T* = 300, 390, 507, 659, 857, 1114, 1448, 1882, and 2447 K. This temperature spacing was suggested by Beauvais et al.^38^ to ensure good overlap between successive temperatures. The positions of framework atoms (Si, Al, and O) were fixed in all simulations. For zeolites with Si/Al = 1 (e.g., 4A), Al atoms were distributed in an alternating pattern with Si atoms (···–Si–O–Al–O–Si–O–Al–O–···) to adhere to Lowenstein’s rule (no –Al–O–Al– linkage). For zeolites with Si/Al > 1 (e.g., NaX), Al atoms were randomly distributed using the recipe discussed by Findley et al.^39^

### 2.6 Grand Canonical Monte Carlo Simulations

Single-component adsorption isotherms of water in all zeolites were simulated using RASPA.^37^ Although including framework vibrations can be important in making accurate adsorption predictions in some porous materials like MOFs,^40,41^ these effects are small for zeolites.^42–45^ In this work, GCMC simulations were performed with all framework atoms assumed to be rigid but allowing extra-framework cations to move. Sodalite cages in LTA (zeolite A) and FAU (zeolites X and Y), which are known to be inaccessible to adsorbates, were blocked in all GCMC simulations. When a unit cell had a lattice parameter shorter than 24 Å in any direction, the cell was expanded enough to satisfy the minimum image convention. To ensure well-converged results,^39^ the production part was started after 5 × 10^4^ initialization cycles and thermodynamic properties were sampled over 10^5^ cycles.

### 2.7 Molecular Dynamics Simulations

Classical molecular dynamics were carried out using the LAMMPS code.^46^ Simulations were performed in the *NVT* ensemble using a Nosé–Hoover thermostat^47,48^ with a chain length of 6 and a relaxation time of 0.1 ps. The velocity-Verlet algorithm was used to integrate the equations of motion with a time step of 1 fs. The Ewald method^49^ was used to compute long-range electrostatic interactions with a precision equal to 10^–6^. A cutoff of 11 Å was set for both electrostatic and van der Waals interactions. In all MD simulations, framework atoms (Si, Al, and O) were fixed to their experimental positions, and only cations and adsorbed molecules were allowed to move. Each MD simulation was equilibrated for 1 ns, followed by a 40 ns production period. Self-diffusion constants were derived through a linear fit of time evolution of mean-squared displacement (MSD) to Einstein equation, ⟨*r* ^2^⟩ = 2*dD*_s_ *t* (*r* is adsorbate displacement, *t* is time, *d* is dimensionality of the system, and *D*_s_ is self-diffusion coefficient).^50^ Each diffusion constant was averaged over data from ten independent *NVT* MD runs with different initial velocity distributions.

## 3 Results

### 3.1 Prediction of Water Adsorption in Cationic Zeolites

#### 3.1.1 Adsorption Isotherms

Wang^7^ carried out measurements of water adsorption isotherms in 3A (K-LTA) and 4A (Na-LTA) crystals over a pressure range of 10^–3^ to 10 kPa and temperatures from 25 to 250 °C (298 to 523 K). In these experiments, 4A zeolite contained only Na with Si/Al = 1 while 3A was partially exchanged (58 mol % Na and 42 mol % K) with Si/Al = 1.1.

Simulation boxes of 4A and 3A were constructed to match these chemical compositions. The experimental study focused on water adsorption in crystals, not pelletized zeolites or beads containing binders encountered in most commercial samples of 3A and 4A zeolites. Simulated adsorption isotherms using CCFF are in good agreement with Wang’s measurements (see Figures 1 and 2) over the full pressure range of the experiments for all temperatures. We emphasize that the CCFF results shown here and in later figures are predictions made using our force field and not results that have been fitted in any way to the experimental data. A comparison to experimental data from Rakhmatkarieva et al.^51^ measured at in 4A at 303 K, which is in reasonable agreement with the experimental data from Wang et al. at 298 K, is shown in Figure S7.

**Figure 1:**
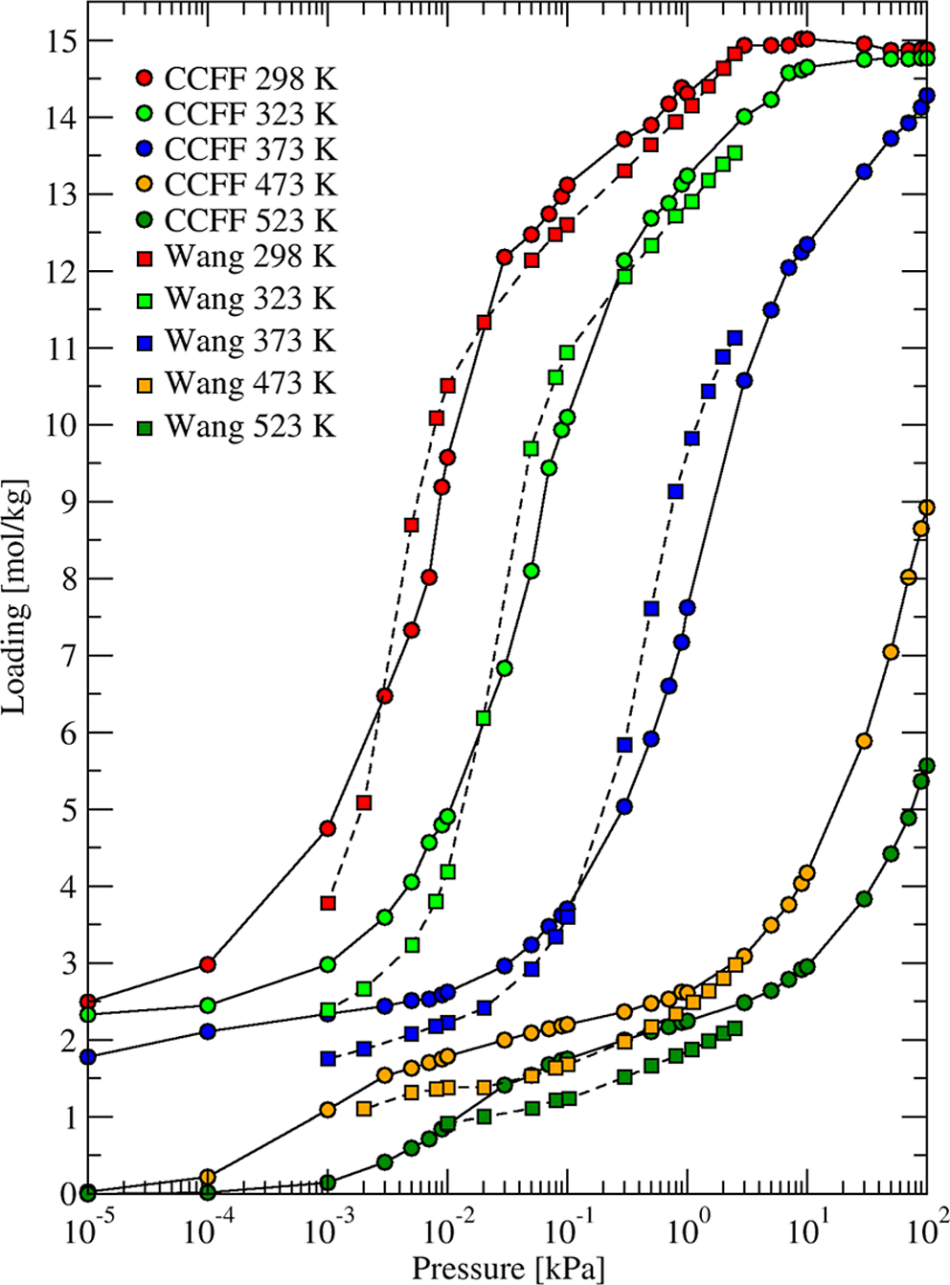
Simulated (circles and solid curves) and experimental^7^ (squares and dashed curves) adsorption isotherms of H_2_O in 4A zeolite at different temperatures.

**Figure 2:**
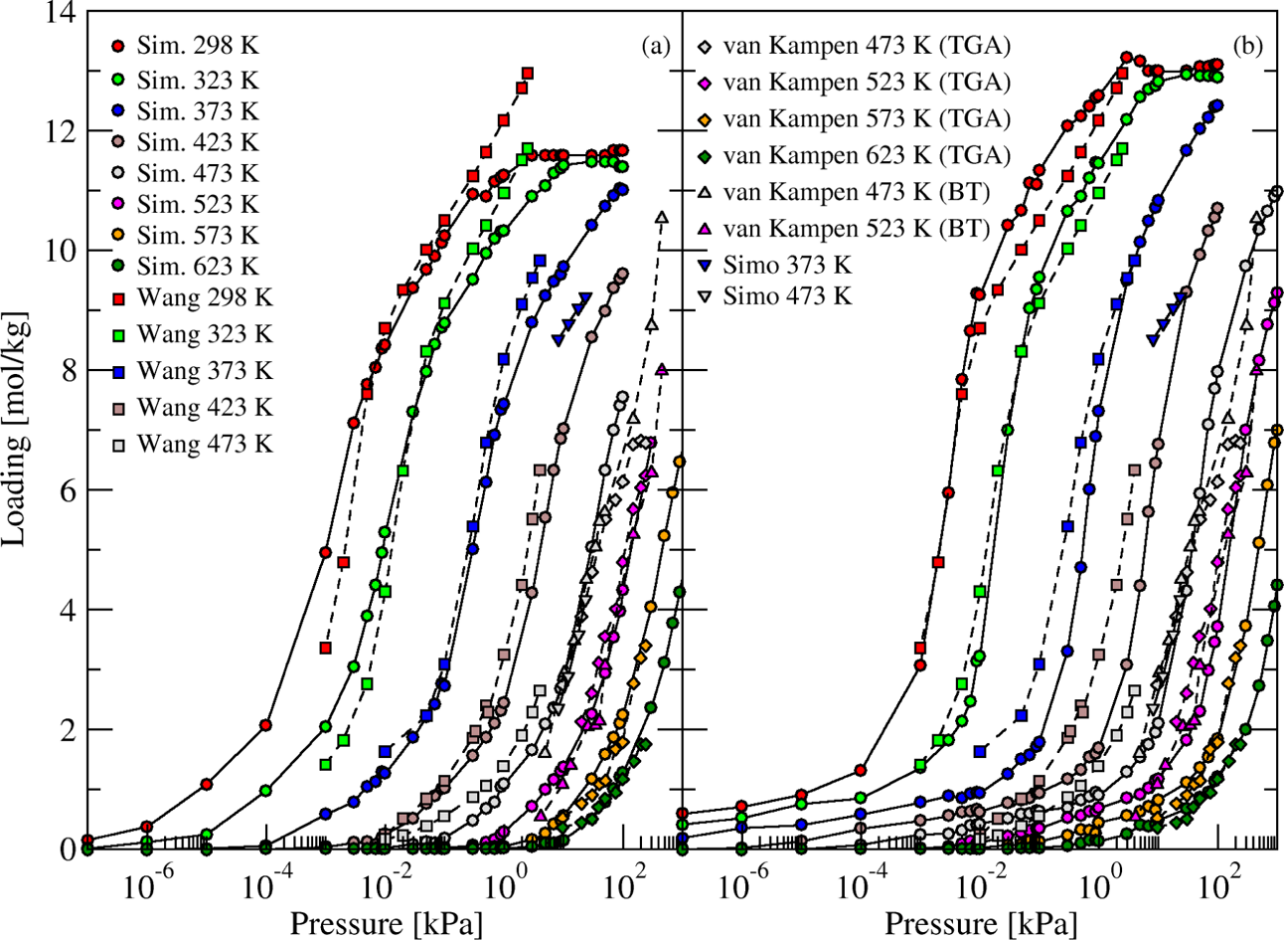
Simulated (circles and solid curves) and experimental^7,52,53^ (squares, diamonds, and triangles and dashed curves) adsorption isotherms of H_2_O in (a) fully (KA) and (b) partially exchanged (KNaA) zeolite 3A structures at different temperatures.

It is clear from Figures 1 and 2 that 4A zeolite has a higher capacity for water than 3A zeolite. The simulated working capacity at ambient temperature of 3A was 11.5% lower than 4A, in agreement with experimental findings. As the accessible volume remains constant in rigid LTA zeolites, the steric encumbrance introduced by K cations with larger ionic radius reduced the maximum number of water molecules adsorbed in 3A compared to 4A. Both simulated and measured isotherms indicated that the water capacity of 3A at 10^–2^ kPa was negligible at 473 K (200 °C) while 4A still had a little more than 1 mol/kg water at these conditions. This explains why 4A needs higher regeneration temperatures compared to 3A for similar dehydration conditions.

Various adsorption models were tested for water adsorption isotherms in 3A and 4A, and the triple-site Langmuir model described the experimental data well over the entire range of measured concentrations for all temperatures. This is consistent with cationic Linde-type structures having three different sites (6-ring, 8-ring, and 4-ring cations) for water adsorption. Zhang et al.^54^ inferred the existence of these three sites for water adsorption in 3A using infrared spectroscopy coupled with multivariate data analysis. However, our GCMC simulations showed that the small number of Na cations occupying 4-ring positions (1 Na cation per super cage) were displaced to form doubly occupied 8-ring sites in 4A zeolite. This is still consistent with the triple site adsorption model because the energetics of water interaction with single occupied and double occupied 8-ring sites are different.

Simulated adsorption isotherm of water in 3A zeolite are also in good agreement with experimental data measured by van Kampen et al.^52^ obtained from thermodynamic analysis and integrated breakthrough curves at 200–350 °C and 5–450 kPa (see Figure 2). Data from breakthrough experiments carried out by Simo et al.^53^ on water adsorption at high temperatures in 3A with different particle sizes are also in a good agreement with simulated isotherm using CCFF (see Figure 2). As in previous work with the CCFF,^13,14^ the good agreement between simulated isotherms and experimental data supports the conclusion that describing the adsorbate–zeolite interactions with coupled cluster accuracy leads to quantitatively accurate results.

For 5A (NaCaA) zeolite, the commercially available samples that are usually used in adsorption measurements were reported to have varied degrees of Na exchange.^8,61–63^ To explore the effect of Ca and Na content on water adsorption in 5A, we used 12 different experimental structures of NaCaA with Si/Al = 1 obtained from the ICSD database.^64^ These structures can be grouped in three sets by the number of Ca and Na cations per super cage: 2Ca + 8Na, 4Ca + 4Na, and 5Ca + 2Na, corresponding to 33%, 67%, and 83% Na exchange, respectively. The unit cell volumes of these 12 experimentally reported structures vary from 14,681 to 15,327 Å^3^. The majority of commercial 5A samples used in adsorption studies and 8 of the 12 NaCaA structures available in ICSD have 67% Na exchange (or 4Ca + 4Na per super cage).

The distribution of Na and Ca cations over the available sites in each of the 12 NaCaA structures was determined by using parallel tempering simulations as described above. Ca cations preferred to occupy 6-ring positions in all structures. Na cations were distributed between 6-ring and 8-ring sites in 2Ca + 8Na and 5Ca + 2Na structures and only in 6-ring sites in 4Ca + 4Na structures. The simulated isotherms using CCFF are compared to the experimental data from Wang and LeVan,^8^ who used volumetric and gravimetric apparatus to measure water uptake on 5A spherical beads at temperatures of 0–100 °C. The degree of cation exchange and Si/Al ratio in these samples were not reported. The simulated isotherms based on 5A structures with 4Ca + 4Na per cage are in good agreement with the experimental data for most temperatures, as shown in Figure 3. Isotherms computed using 5Ca + 2Na structures overestimated uptake compared to the experimental data, as shown in Figure S8. On one hand, the higher Ca content favored higher H_2_O uptake considering the strong attractive electrostatic H_2_O–Ca interactions. On the other hand, lower number of cations per cage compared to the two other sets resulted in a larger accessible volume. Isotherms computed using 2Ca + 8Na structures also overestimated uptake compared to the experimental data, as shown in Figure S8.

**Figure 3:**
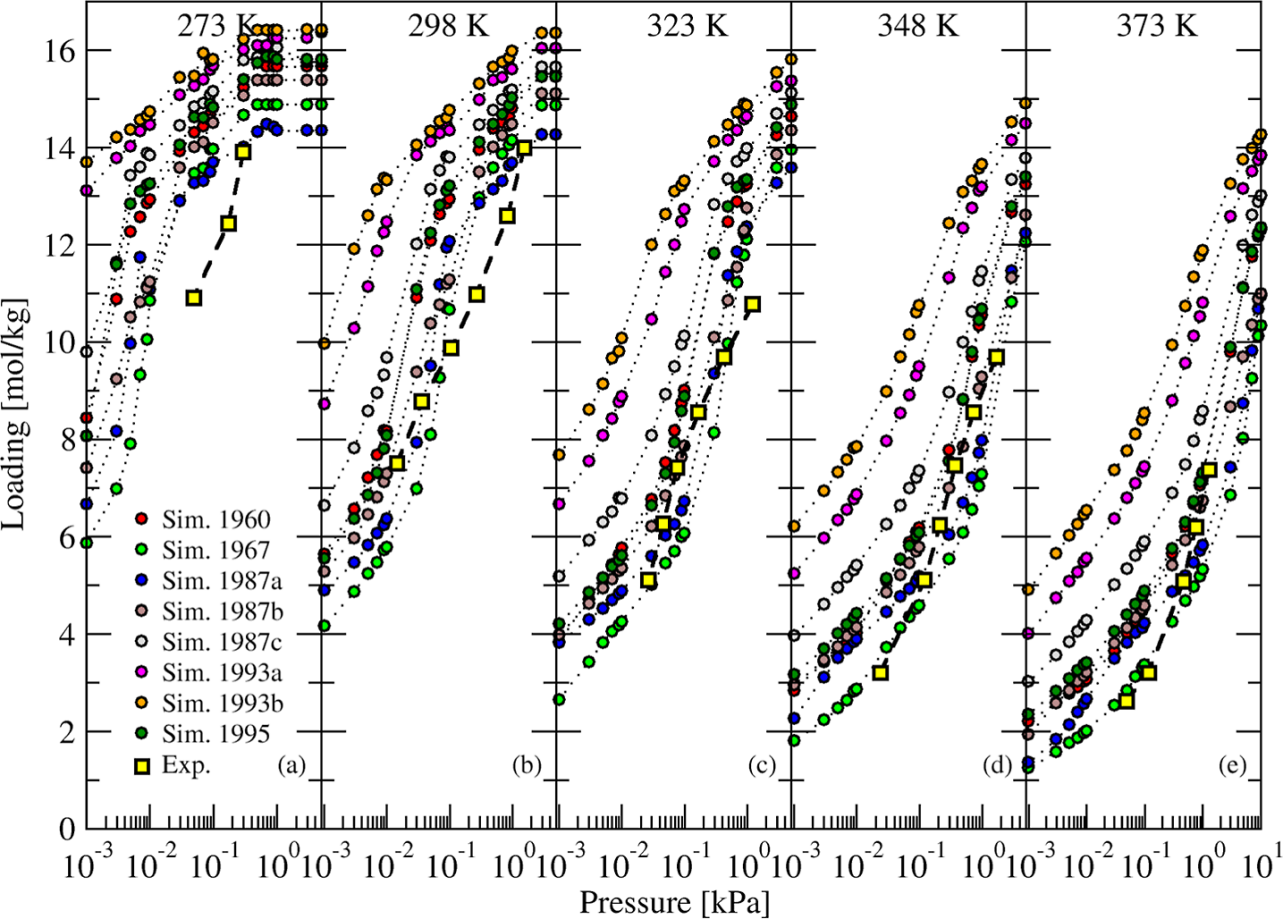
Simulated (circles and dotted curves) and experimental^8^ (squares and dashed curves) adsorption isotherms of H_2_O in 5A zeolite at 5 different temperatures. Eight experimental frameworks^55–60^ of 5A zeolite 4Ca + 4Na per cage were used in simulations.

The exchange of Na with Ca, unlike in 3A with K, is not expected to lower the regeneration temperature compared to that of 4A zeolite. Uptake at 100 °C from both simulated and experimental isotherms was above 2 mol/kg at 10^–2^ kPa. This is due to stronger H_2_O–Ca interaction compared to both H_2_O–Na and H_2_O–K. At ambient temperature and near saturation of water vapor, the simulated uptake in the different 5A structures ranged between 14 and 16 mol/kg, comparable to the experimental value of ∼14 mol/kg. The experimental saturation loading decreased from ∼14 mol/kg at 273 K to ∼7 mol/kg at 373 K. This trend was reproduced by our simulations, with a decrease from 15 ± 2 to 8 ± 2 mol/kg. At ambient temperature and for pressures of 10^–2^ to 1 kPa, the working capacity of 5A (∼7 mol/g) almost doubled compared to 3A (∼3.5 mol/kg) and 4A (∼4 mol/kg). Because the unit cell parameter of LTA structures (3A, 4A, and 5A) is comparable, the accessible volume of 5A increased when part of Na cations was substituted by Ca cations with a ratio of 2:1, leading to less steric hindrance compared to 3A and 4A.

Several experimental studies^8,65–71,73–81^ reported equilibrium adsorption isotherm of water on NaX (13X) zeolite. These studies can be grouped based on the type of adsorbent used in their measurements, i.e., crystalline or pelletized. Isotherms obtained from pelletized adsorbents showed a rapid increase in the uptake as water vapor partial pressure approached the saturation value, leading to capillary condensation within mesopores and macropores formed by the clay binder in the pellets.^65,71,81,82^ The increase in the adsorption associated with condensation in pelletized adsorbents was not observed in isotherms obtained from crystalline adsorbents. Most of these experiments did not extend to high pressure where macropore condensation occurs and only one isotherm showed steady increase in the water uptake up to saturation pressure.^71,81^

The experimental studies on NaX can be split into two sets based on their capacity for water adsorption. The first set, which includes most of the studies described above, displayed a capacity of ∼18 mol/kg at 1 kPa and ambient temperature. The second set showed a significantly lower capacity of ∼15 mol/kg under the same conditions. The water adsorption capacity predicted by our simulations (see Figure 4) was ∼18 mol/kg, in agreement with the majority of experimental results from the literature.^8,65–71,73–81^ The difference in capacity between these sets of experiments was attributed to differences in the formulation of adsorbents.^81^ Aldrich^65,69,75^ and Zeochem ZEOX^77^ pellets consistently led to low-capacity isotherms compared to the majority of isotherm in the literature. Most experiments with these and similar pellets included 15–20% binder mass fraction.

**Figure 4:**
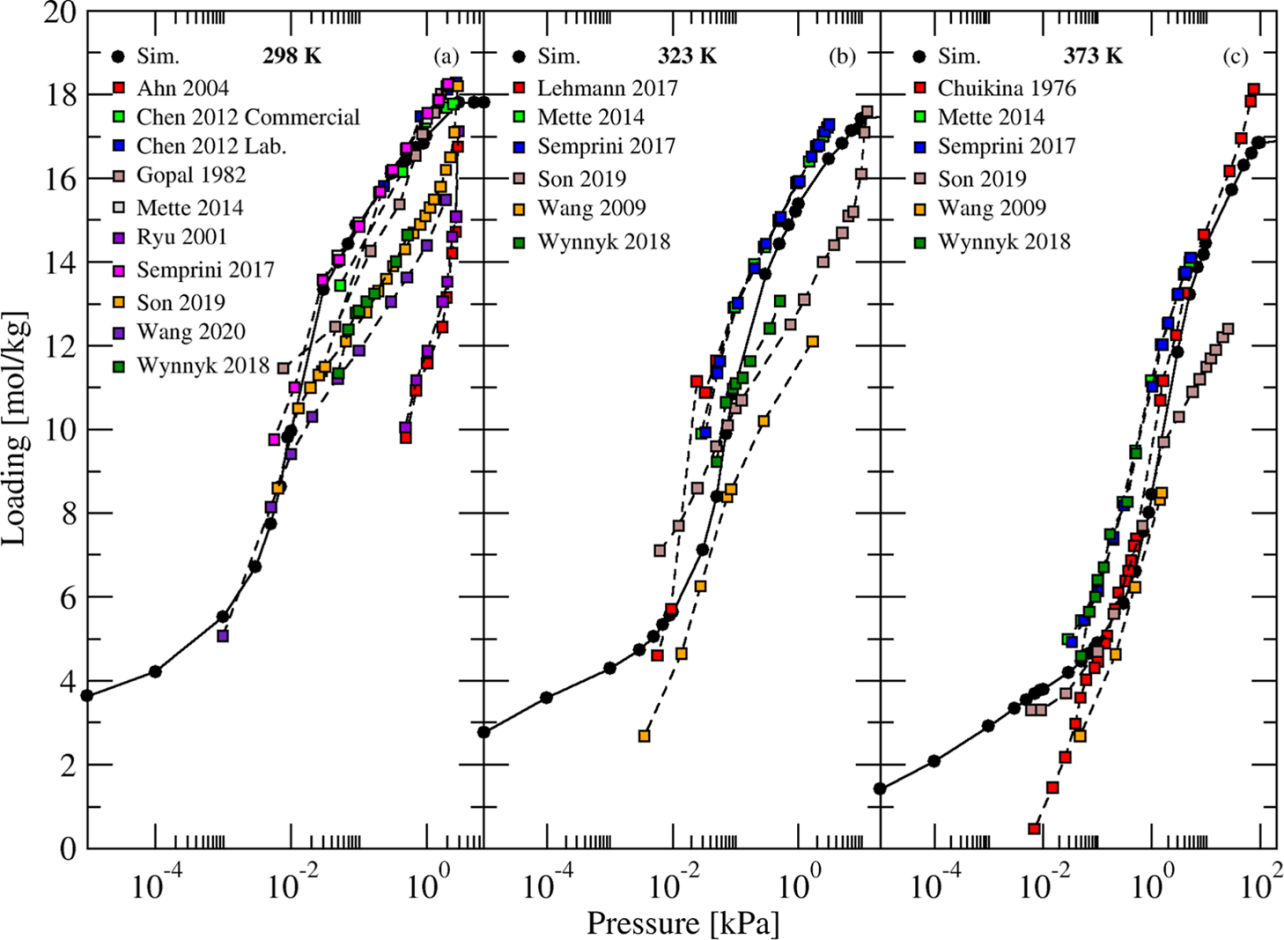
Simulated (circles and solid curves) and experimental^7,8,65–73^ (squares and dashed curves) adsorption isotherms of H_2_O in NaX zeolite at different temperatures.

Son et al.^71,81^ showed that an adequate activation temperature is necessary to remove all residual water and obtain accurate measurements with NaX. They compared their data using NaX activated at 350 °C to low-capacity isotherms obtained after activation at 175 °C by Wang and LeVan.^8^ This is consistent with Brandani and Ruthven,^83^ who demonstrated that an activation temperature of at least 350 °C was needed to completely remove residual water in their study of the effect of H_2_O on CO_2_ adsorption in NaX. The elimination of sources of bias that led to the scatter in experimental adsorption capacity and behavior of isotherms of water in NaX is challenging. The use of our CCFF model in combination with GCMC simulations is a useful tool to achieve accurate prediction of adsorption properties of water in NaX zeolite.

To illustrate the role of both residual water and binder present in most experiments, we compared our theoretical predictions to two distinct experimental cases (see Figure 5). In the first, Chuikina et al.^73^ used laboratory-made and binder-free NaX samples activated at 400 °C for 100 h, allowing thorough removal of the residual water. In terms of composition, absence of binder, and traces of water, this experimental system is equivalent to the zeolite used in our GCMC calculations. In the second case, Wang and LeVan used NaX beads with 18% mass of binder and an activation temperature of 175 °C in a vacuum overnight. These experiments have two sources of uptake measurement uncertainty if the results are interpreted as adsorption isotherms for “pure” NaX, as pointed out by Son et al.^71,81^ Our simulated isotherms at 298 and 373 K are in a good agreement with data from Chuikina et al.^73^ (Figure 5a). Isotherms from Wang and LeVan^8^ showed reduced water uptake for all temperatures (273, 295, 323, 348, and 373 K) compared to our simulations (Figure 5b). At ambient temperature, their loading was consistently lower by 0.5–3 mol/kg than both our simulations and the experiments of Chuikina et al.^73^ This comparison further validates the accuracy of our CCFF model for water in Na-exchanged zeolites. Some systematic deviation exists between our simulated isotherms and the experimental results of Chuikina et al.^73^ at the lowest pressures explored in those experiments. Making high precision experimental measurements in this pressure range (10^–3^ to 10^–1^ kPa, i.e. 10^–5^ to 10^–3^ atm) is extremely challenging, so we lean toward concluding that the change in slope of the simulated isotherms at these pressures and below is correct but that reaching true equilibrium experimentally at these conditions was not achieved by Chuikina et al.^73^

**Figure 5:**
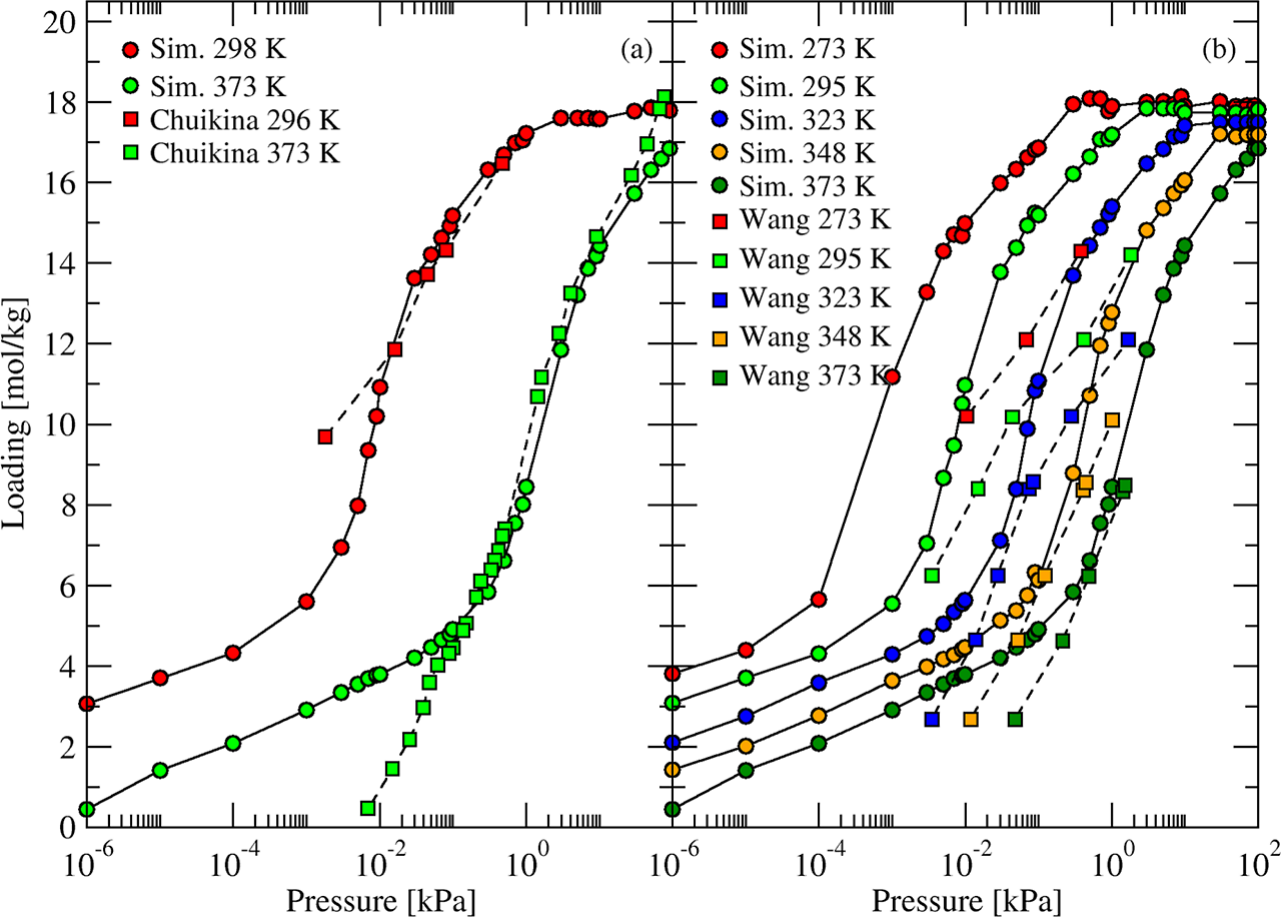
Comparison between simulated (circles and solid curves) and experimental (squares and dashed curves) adsorption isotherms H_2_O in (a) binder-free and (b) binder-containing samples of NaX at different temperatures.

Ammouli et al.^84^ used a manometric method to measure the water uptake on K-exchanged zeolite X after activation at 300 °C under vacuum for 24 h. The adsorbent was prepared by cationic exchange from commercial NaX with a Si/Al ratio of 1.23. The chemical composition of the final product was determined using X-ray fluorescence as K_81_NaH_4_[Si_106_Al_86_O_384_]. Substitution of Na cations by protons during cation exchange has been reported for other monovalent cations by Dzhigit et al.^85,86^ Simulated water adsorption isotherms using the CCFF showed good agreement with the experimental data (see Figure 6). The divergence of capacity in the experimental data near the water vapor saturation pressure was probably caused by capillary condensation occurring in pores formed by the binder.

**Figure 6:**
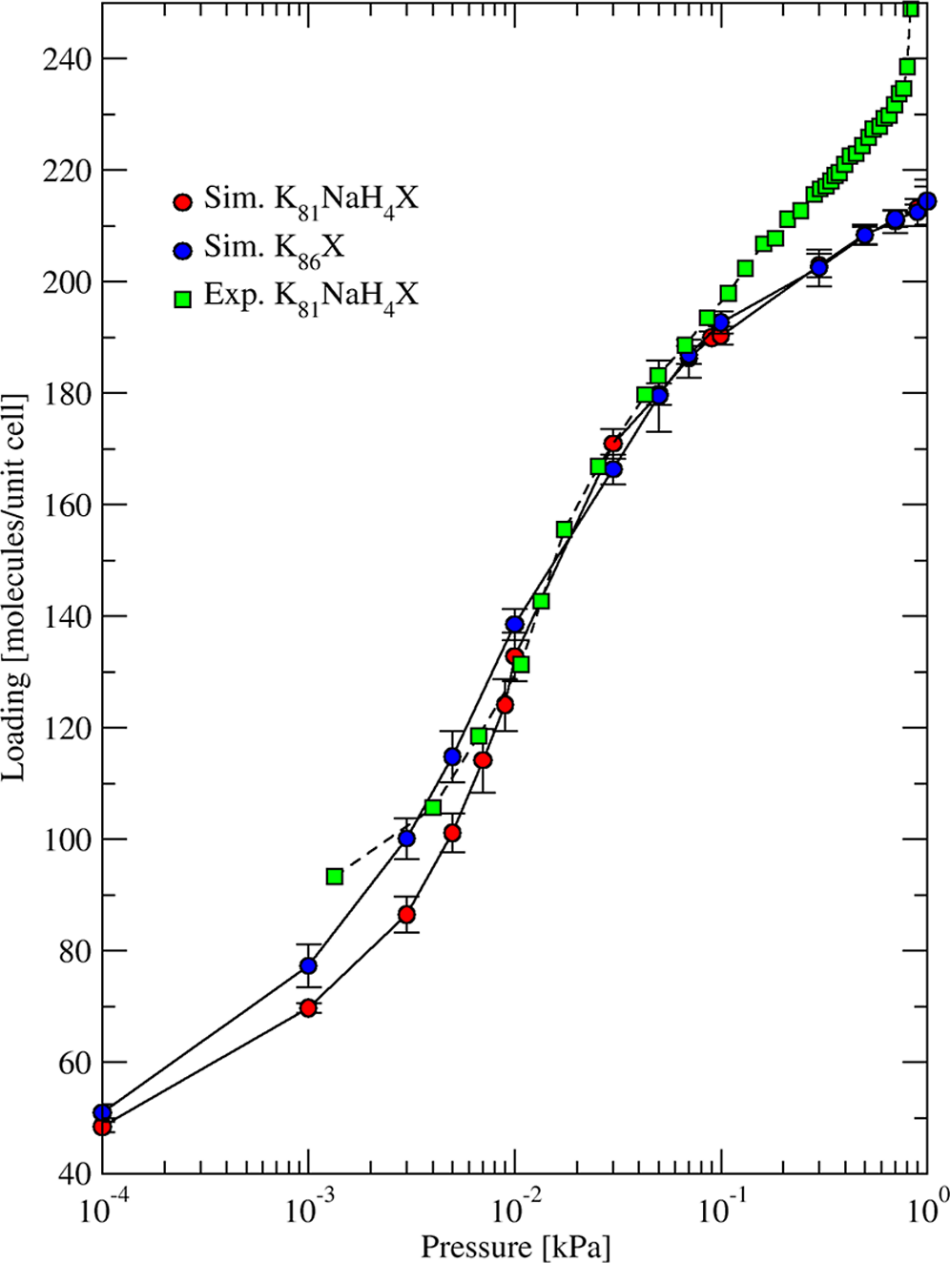
Simulated (red and blue circle and solid curves) and experimental^84^ (green squares and dashed curve) adsorption isotherms of H_2_O in K-exchanged zeolite X at 303 K.

To determine the effect of Na cations and protons on water adsorption in K-exchanged zeolite X (K_81_NaH_4_X), we performed simulations on pure K zeolite X (K_86_X). Because of the small number of Na cations and protons, the difference between the two simulated isotherms is negligible. Using a combination of atomic-scale simulation techniques, Ammouli et al.^84^ argued in favor of an adsorption scenario where water molecules preferably occupy sodalite cages and only start to fill the super cages at high pressures. Our results disagree with this description since access to sodalite cages was blocked during our GCMC simulations, but the resulting adsorption isotherm is still in good agreement with the experimental data.

Dzhigit et al.^85^ reported adsorption isotherms of water in LiNaX, NaX, KNaX, RbNaX, and CsNaX at 23 °C. The zeolites were evacuated at 400 °C for ∼100 h. The different zeolites were prepared from NaX by means of ion exchange with aqueous solutions of the corresponding salts. All zeolites were subject to various degrees of decationization, and their Si/Al ratio was determined to be in the range of 1.31–1.38. Based on experimental observations^73,84,85^ and to maintain charge neutrality of these MX zeolites, the missing cations were replaced by protons in our GCMC simulations. The chemical compositions of different MX zeolites are summarized in Table 5.

**Table 5:** Chemical Composition of MX Zeolites (M = Li, Na, K, Rb, Cs) Used in GCMC Simulations

zeolite	Si/Al	degree of decationization [%]	Si	Al	M	Na	H	O
LiX	1.34	1	110	82	50	31	1	384
NaX	1.37	6	111	81	76		5	
KX	1.31	8	109	83	55	21	7	
RbX	1.31	8	109	83	52	24	7	
CsX	1.34	8	110	82	45	30	7	

The simulated and experimental adsorption isotherms in Figure 7 are in good agreement. A considerable amount of water was adsorbed in all zeolites even at low pressures, and about 50% or more of maximum capacity was achieved with pressures as low as 0.1 kPa. The water adsorption capacity, per kilogram of zeolite, decreased by ∼39% as the cation size increased from Li to Cs. This is correlated with a decrease in accessible volume of 42% measured by Dzhigit et al.^85^ This trend is also consistent with the decrease in the temperature required for complete desorption or dehydration of zeolite X with monovalent cations reported by Joshi et al.^87^ (700, 607, 583, and 574 K for NaX, KNaX, RbNaX, and CsNaX, respectively).

**Figure 7:**
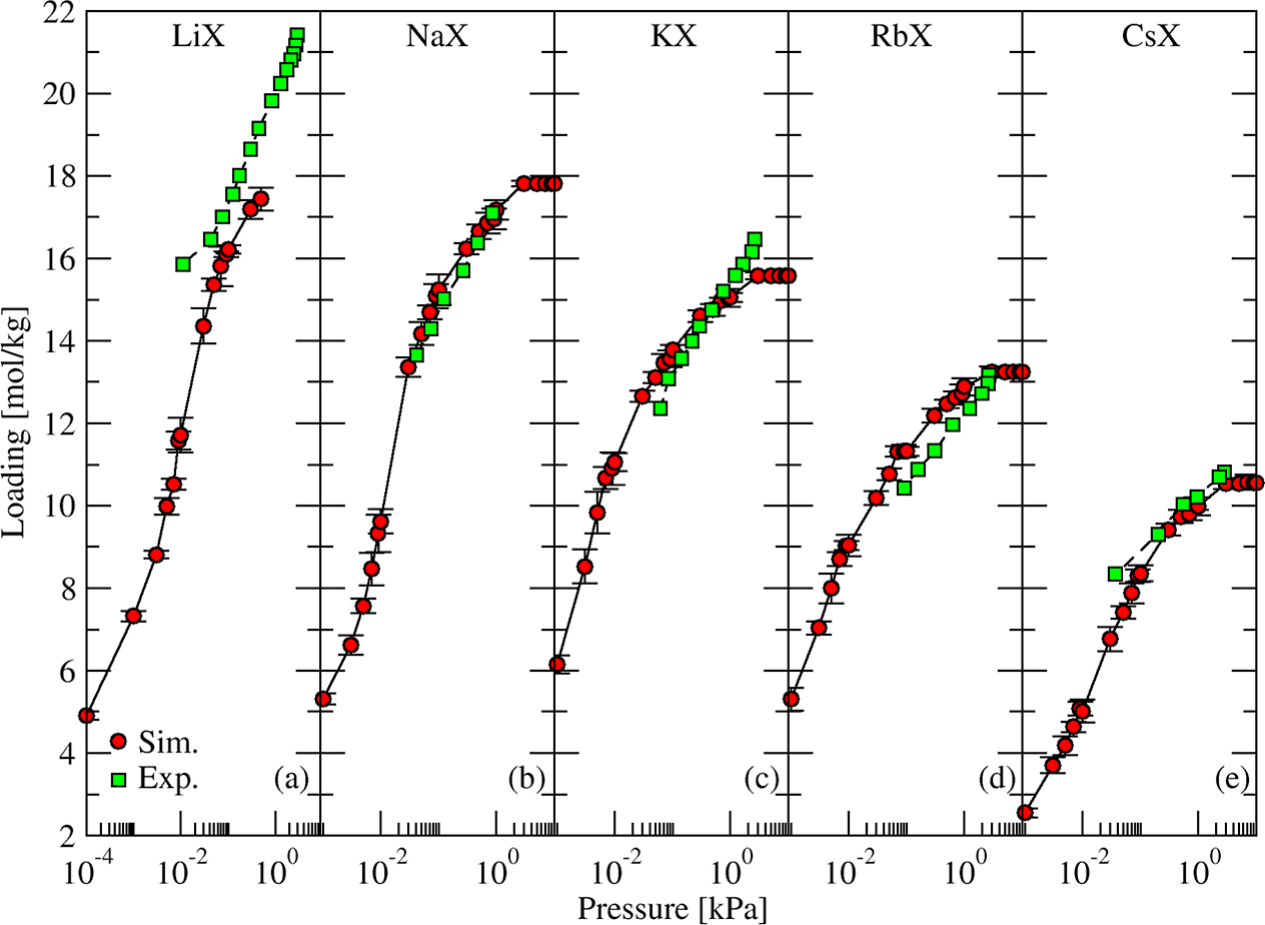
Simulated (red circles) and experimental^85,86^ (green squares) adsorption isotherms of H_2_O in MX zeolites (M = Li, Na, K, Rb, and Cs) at 296 K.

#### 3.1.2 Heats of Adsorption

Information obtained from the heat of adsorption is important to better understand isotherms and the energy required to regenerate samples in TSA processes. Computationally, heat of adsorption can be either directly obtained from GCMC simulations at a given temperature or using the following Clausius–Clapeyron equation applied to isotherms collected at different temperatures,

7 $$q_{st}=-R{\left[\frac{\partial \ln P}{\partial \left(\frac{1}{T}\right)}\right]}_{n}$$

where *R* is the gas constant (8.314 J/mol/K). To use the Clausius–Clapeyron equation, adsorption isosteres were calculated based on adsorption isotherms. To this end, values of pressure (*P*) corresponding to a series of adsorbed amounts (*n*) were determined via either an analytic model or interpolation. The values of *q*_st_ were calculated from the slopes of these adsorption isosteres. Wang fitted experimental adsorption isotherms of H_2_O in 3A and 4A using a triple-site Langmuir model to determine the adsorption isosteres.^7^ In this work, we used the Piecewise Cubic Hermite Interpolating Polynomial (PCHIP)^88^ procedure via Matlab to determine the adsorption isosteres. We found that this procedure suffered less residual error compared to fitting with triple-site Langmuir models, especially at low loadings. For a more direct comparison between simulation and experiment, we used the experimental adsorption isotherms from Wang^7^ to determine experimental adsorption isosteres and *q*_st_ with the PCHIP procedure.

For the 4A zeolite, the heat of adsorption at infinite dilution obtained from GCMC simulations is ∼110 kJ/mol for all temperatures. This is a clear indicator of strong water–framework interactions and explains why a high temperature is needed in order to activate or regenerate 4A samples. Heats of adsorption at all temperatures dropped significantly from ∼110 kJ/mol to 55–75 kJ/mol for loadings between 0 and 2 mol/kg. This suggests that the number of high energy sites available for the adsorption of water is not large. Heats of adsorption showed some temperature dependence for water loadings of 3–10 mol/kg, but at all temperatures, the values converged to values between 50 and 60 kJ/mol as the saturation loading was approached. When the available experimental data and simulated adsorption data were analyzed with the Clausius–Clapeyron method used in the same way for each data set, the resulting heats of adsorption are in good agreement (see Figure 8).

**Figure 8:**
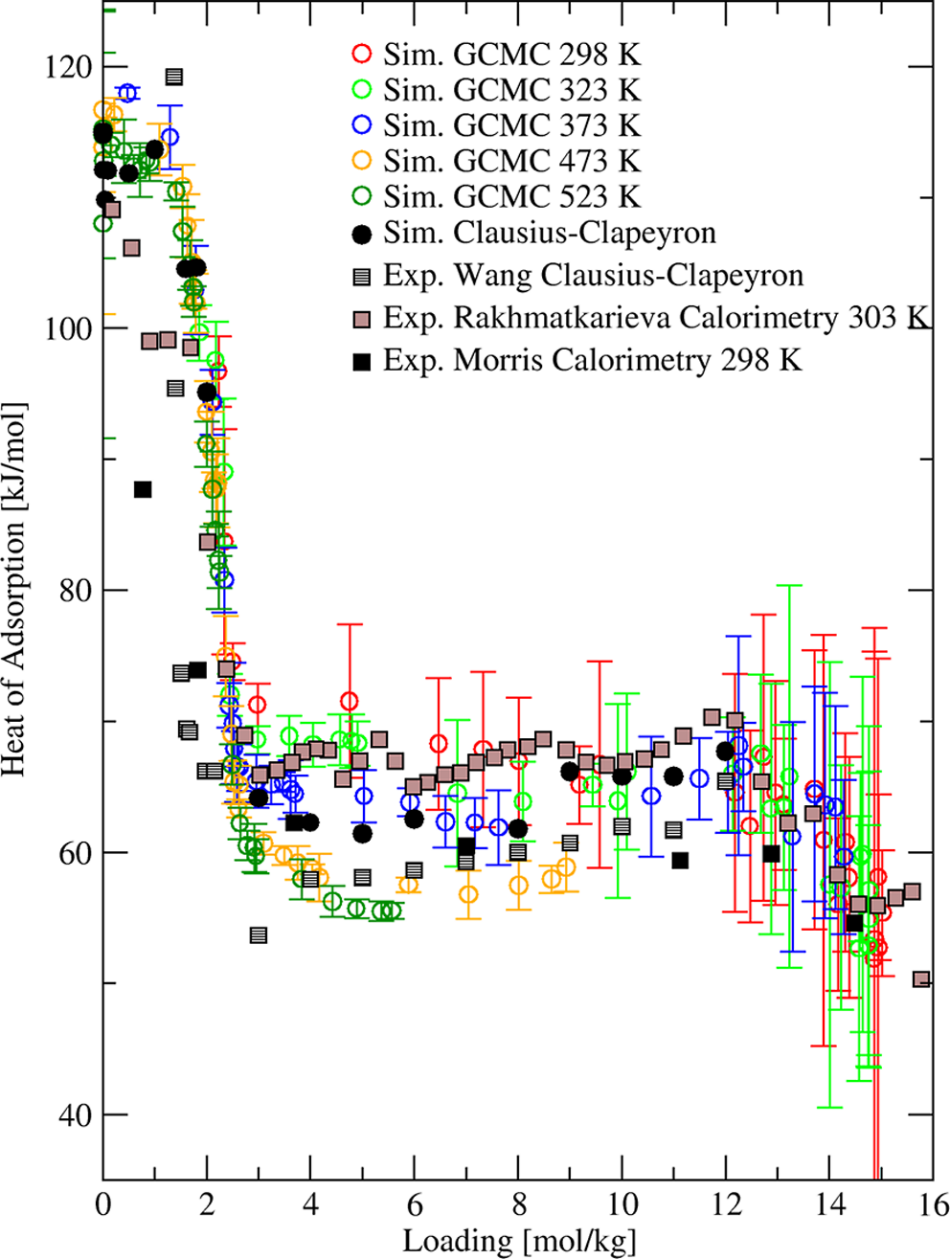
Simulated (circles) and experimental^7,51,89^ (squares) heat of adsorption of H_2_O in 4A zeolite at different temperatures.

An alternative to using the Clausius–Clapeyron approach to determine heats of adsorption is to make direct measurements with adsorption calorimetry. Rakhmatkarieva et al.^51^ used a differential molar adsorption calorimetric method to study the heat of adsorption of water in synthetic binder-free NaA zeolite. Their samples were pumped under high vacuum at 450 °C for 10 h before data were collected at 303 K. This sample treatment is necessary to achieve complete water removal as demonstrated above for NaX based on our simulation results and experimental observations.^71,81,83^ Another calorimetric measurement of isosteric heat of water adsorption was carried out by Morris^89^ at 298 K. Our GCMC predictions are in very good agreement with both sets of measurements, as shown in Figure 8.

To describe the heat of adsorption of water in 3A, we performed two sets of simulations for temperatures of 298–473 K, one using a pure K-LTA structure and the other using the chemical composition (KNaA with Si/Al = 1.1) reported by Wang.^7^ For water loadings of 1.5 mol/kg and higher, heats obtained from GCMC simulations at different temperatures and using Clausius–Clapeyron equation using mixed cation KNaA structure are in better agreement with the experimental data compared to the pure potassium LTA structure (see Figure 9). The slight decrease in heat of adsorption observed experimentally at loadings higher than 9 mol/kg is also reproduced by simulations in KNaA and not KA. The heat of adsorption of water at dilute loadings was much higher in the Na-containing 3A structure compared to K-only 3A. This observation is associated with high energy sites involving Na cations, similar to 4A zeolite.

**Figure 9:**
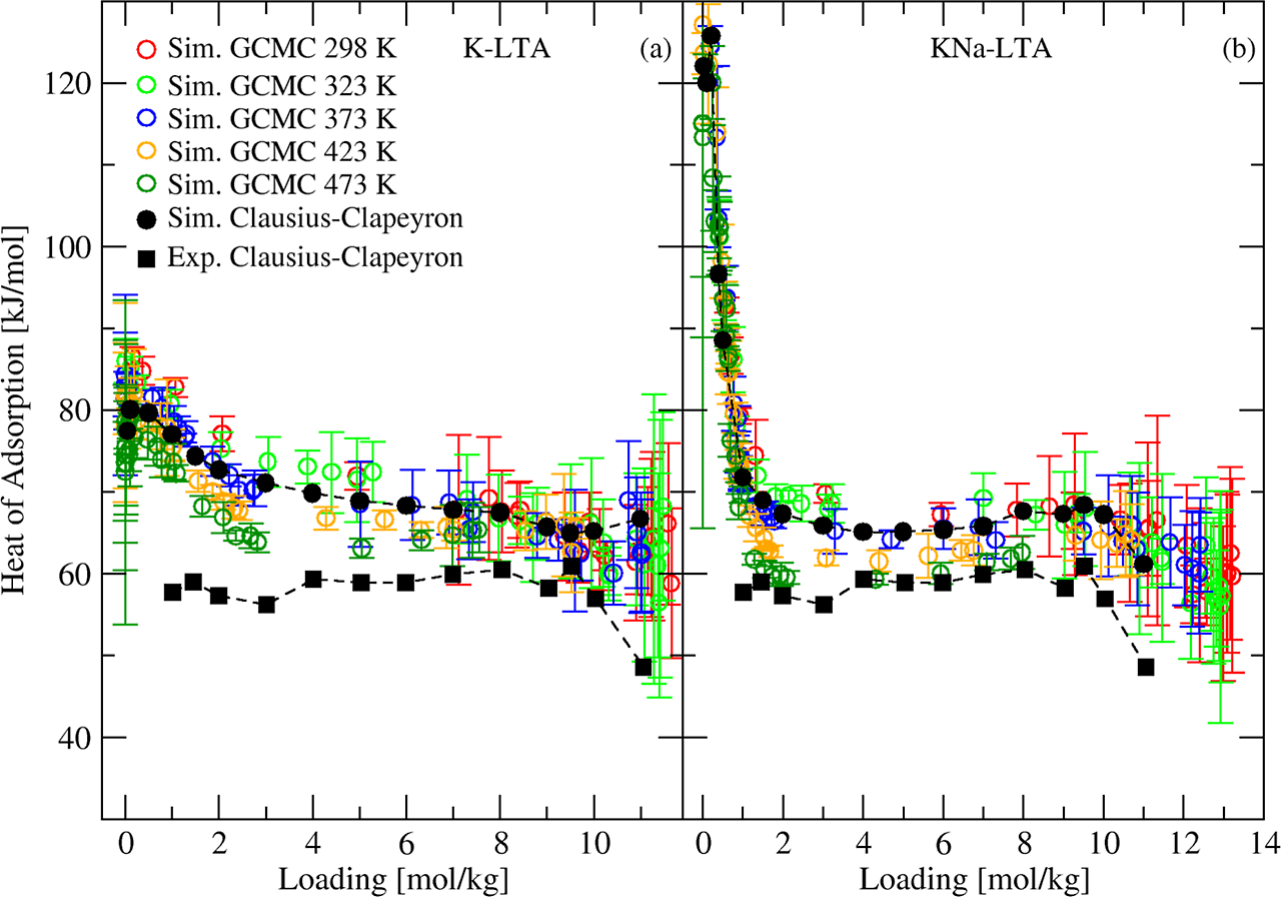
Simulated and experimental^7^ heat of adsorption of H_2_O in (a) K-LTA (3A, Si/Al = 1) and (b) KNa-LTA (Si/Al = 1.1) zeolites at different temperatures.

Several experimental studies^67,73,85,90–92^ reported heats of adsorption of water in NaX zeolite using calorimetric measurements. Similar to 3A and 4A zeolites, the heat of adsorption closer to zero loading reached high values (in the range of 80–90 kJ/mol) before displaying a sharp decrease to ∼60 kJ/mol for loadings between 0 and 5 mol/kg, as shown in Figure 10. This low loading region corresponds to water adsorption on cationic positions characterized by strong water–framework interactions. With increasing uptake, water molecules started to fill up supercage space and heat of adsorption decreases further from ∼60 to ∼50 kJ/mol as the saturation loading was approached. Our heat of adsorption data simulated at 298 and 373 K showed the same trend observed in the experiment with a sharp decrease between 0 and 5 mol/kg followed by a flattening to the saturation loading, as shown in Figure 10.

**Figure 10:**
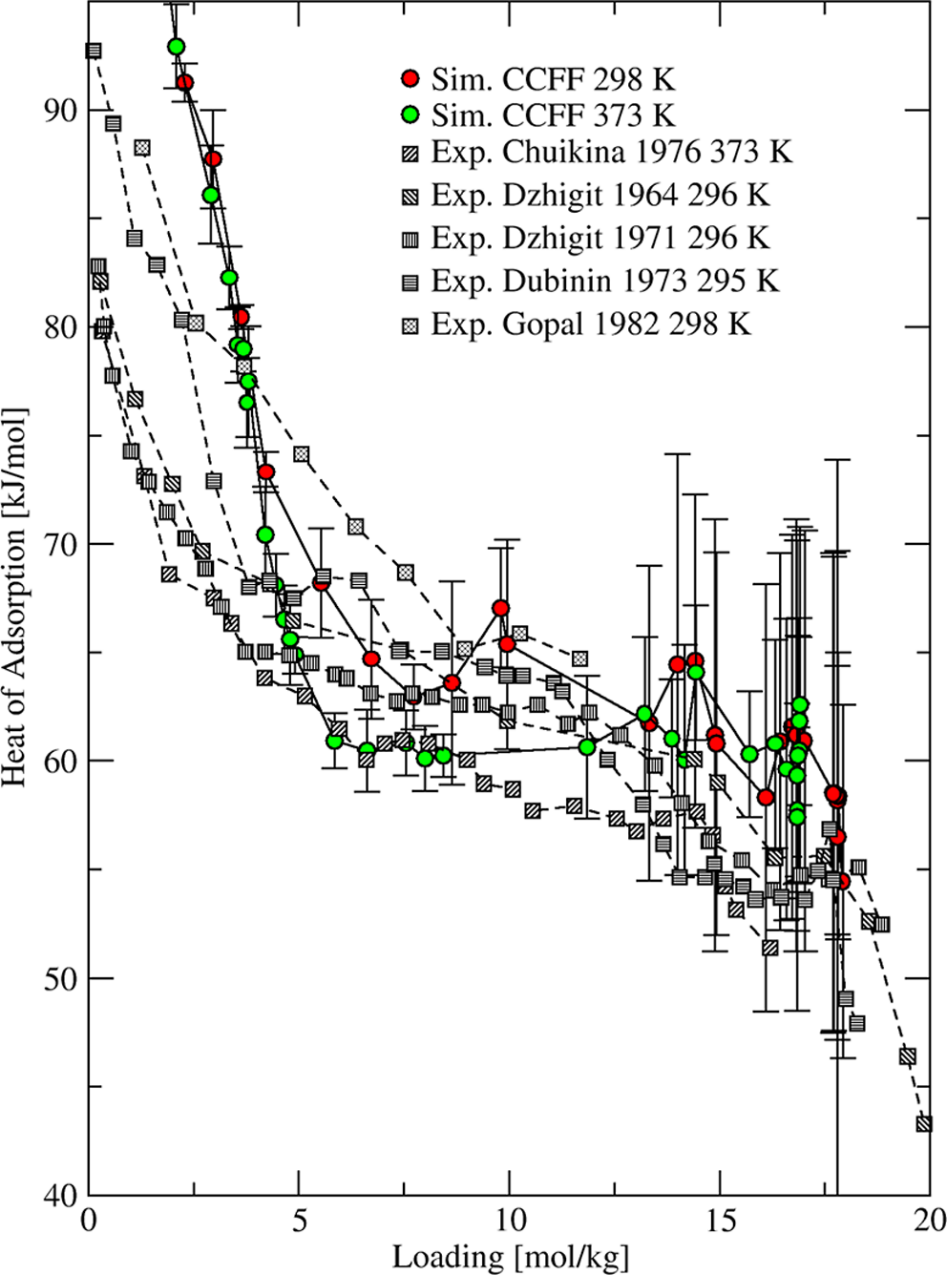
Simulated (circles) and experimental^67,73,85,90,91^ (squares) heats of adsorption of water in NaX zeolite at different temperatures.

Chuikina et al.^73^ measured the heat of adsorption of water in KNaX zeolite at different temperatures from 296 to 373 K. Heats of adsorption obtained from GCMC simulations are in a good agreement with experimental data for all temperatures, as shown in Figure 11. The increase in temperature caused the maxima and minima clearly seen at 296 K to vanish in the heat of the adsorption curves. This was associated by Chuikina et al.^73^ with destabilization of local structures (water molecules bridging cations or water clusters) formed by water in adsorption cavities as temperature increased. These fluctuations were not observed in our simulated data or in experimental measurements for zeolite X of other monovalent cations (Li, Na, Rb, and Cs) done at 296 K by Dzhigit et al.^85,86^

**Figure 11:**
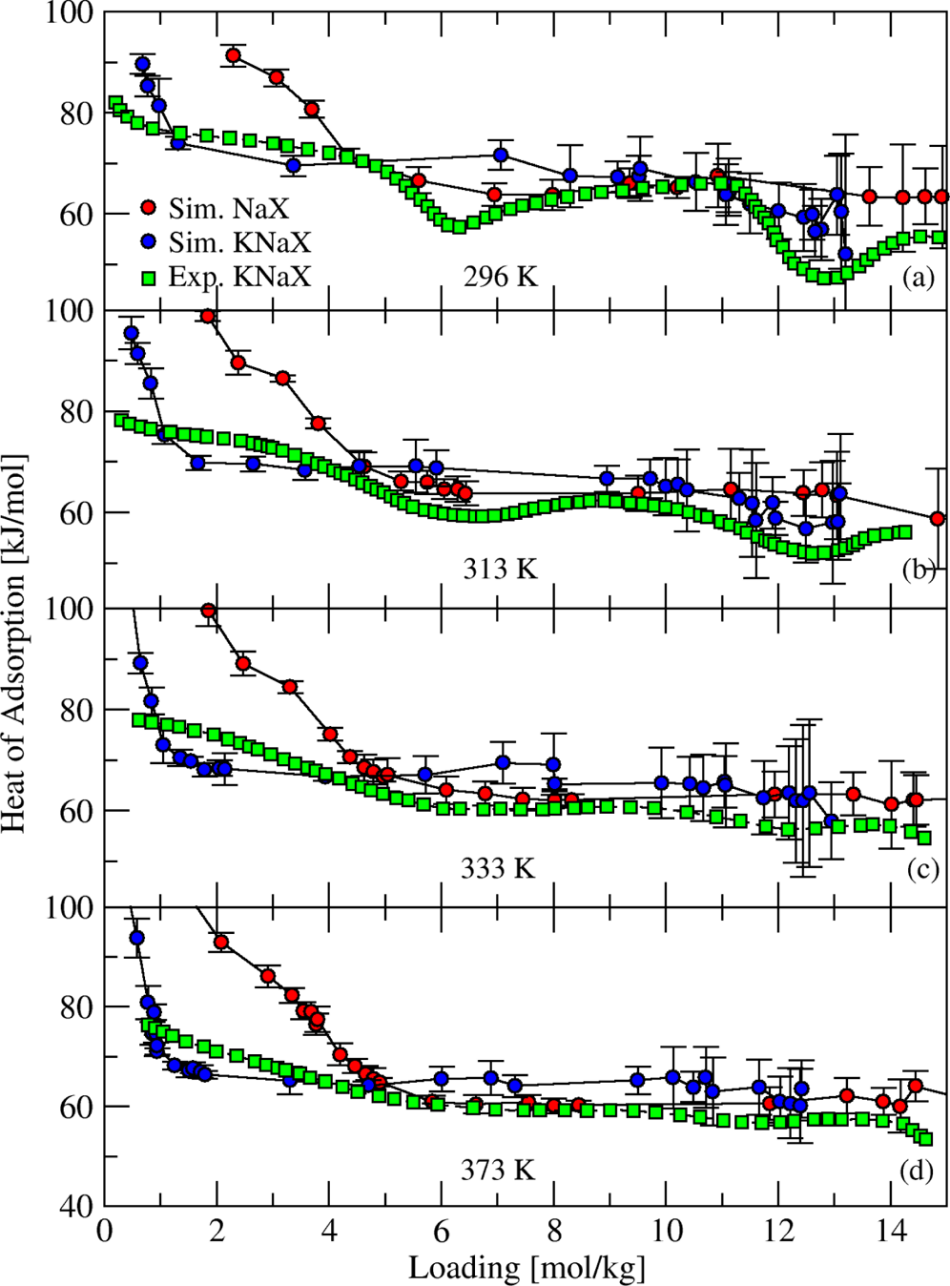
Simulated (red and blue circles and solid lines) and experimental^73^ (green squares and dashed lines) heat of adsorption of H_2_O in NaX and KNaX zeolites at four different temperatures.

To understand the effect of Na partial exchange by K, we included heat of adsorption curves of water in NaX in Figure 11 for all temperatures. For loadings equal to 5 mol/kg and higher, the form and magnitude of the heat of adsorption of NaX and KNaX curves are quite similar. For the low loading region, the partial substitution of Na cations by K resulted in lower heat of adsorption. For example, at ∼2 mol/kg, a decrease of 15–20 kJ/mol was observed between NaX and KNaX. Because water molecules at low loadings predominantly occupy cationic sites, this difference can be explained by weaker water–K interactions compared to water–Na.

Dzhigit et al.^85,86^ reported heats of adsorption of water in zeolites LiNaX, NaX, KNaX, RbNaX, and CsNaX using calorimetric measurements at 23 °C. The chemical compositions of these zeolites are summarized in Table 5. The simulated and experimental heats of adsorption of water in MX zeolites are in good agreement, as shown in Figure 12. The initial region of low loadings up to 4 mol/kg featured a sharp decrease from ∼90 kJ/mol to ∼60 kJ/mol in simulated heats of adsorption for all systems. This characteristic decrease was less pronounced in the experimental data, and values of heat of adsorption near zero loading dropped from ∼85 kJ/mol to ∼65 kJ/mol as the cation size increased. We note that for NaX zeolite, as shown in Figure 10, Gopal et al.^67^ and Dubinin et al.^91^ reported heat of adsorption of water vapor near zero loading of ∼90 kJ/mol, compared to less than 80 kJ/mol from Dzhigit et al.^85,86^ This agreement with the experimental data of Gopal et al.^67^ and Dubinin et al.^91^ for NaX also supports our predictions for zeolite X of other monovalent cations because they all contain between 25 and 38% Na of the total number of cations.

**Figure 12:**
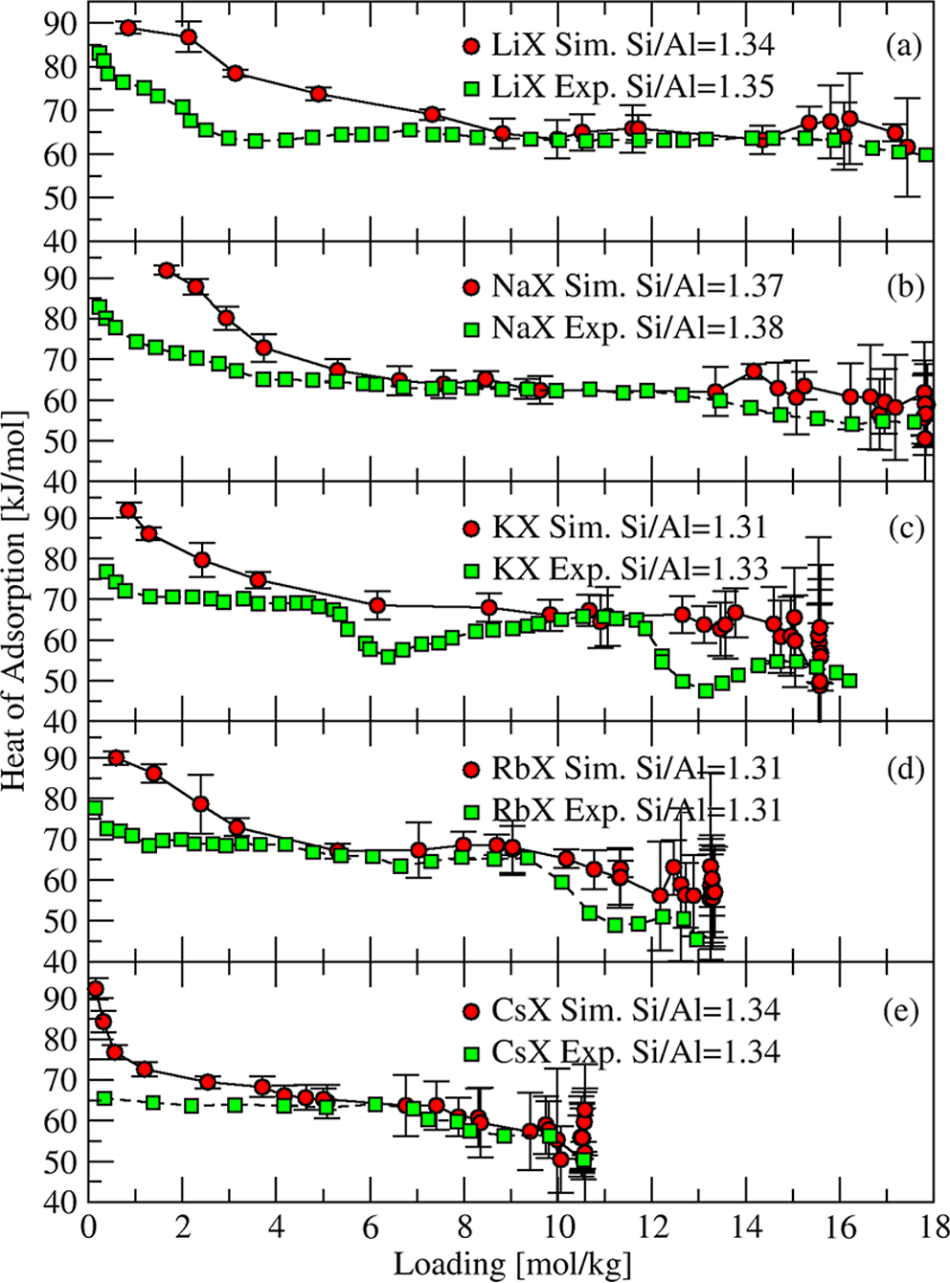
Simulated (red circles) and experimental^85,86^ (green squares) heats of adsorption of H_2_O in MX zeolites (M = Li, Na, K, Rb, and Cs) at 296 K.

### 3.2 Prediction of Water Diffusion in Cationic Zeolites

Because the procedures for developing the CCFF probe in the entire potential energy surface for adsorbed molecules, it is reasonable to expect that this force field can make accurate predictions not only for molecular adsorption but also for molecular diffusion. This outcome has been shown before for CCFF simulations of alkanes in zeolites.^12,13^ Here, we examine the predictions of water diffusion in zeolites using the CCFF.

Several previous experimental^93–97^ and computational^98–101^ studies were devoted to understanding water mobility in cationic zeolites such as 4A. Techniques such as pulsed field gradient nuclear magnetic resonance PFG NMR and quasi elastic neutron scattering QENS were used to determine loading and temperature dependence water diffusivity. However, these microscopic methods yielded overall about one order of magnitude difference in diffusion coefficients. Paoli et al.^93^ interpreted this difference as a reflection of different typical length scales probed by PFG NMR (micrometers) and QENS (nanometers). This difference means that water molecules tracked by PFG NMR would possibly be impacted by transport resistance at the boundaries between adjacent ranges of ideal crystallinity.^93,101^ Our computed self-diffusion coefficients in 4A zeolite, as shown in Figure 13, are overall in good agreement with experimental data reported by Paoli et al.^93^ For low loadings of 5 molecules/cage, the predicted temperature dependence of water is in agreement with QENS measurements. The pattern and non-Arrhenius behavior of PFG NMR data at this loading is likely due to a relatively small crystal size in the experiments, as discussed by Kärger et al. for H_2_O in MFI.^102^ For moderate and high loadings, 15 and 25 molecules/cage, our predicted self-diffusivities are in good agreement with both QENS and PFG NMR data. The computed activation energy of water in 4A at 5, 15, and 25 molecules/cage was 18, 25, and 24 kJ/mol, respectively, compared to 15, 20, and 25 kJ/mol from experiments.

**Figure 13:**
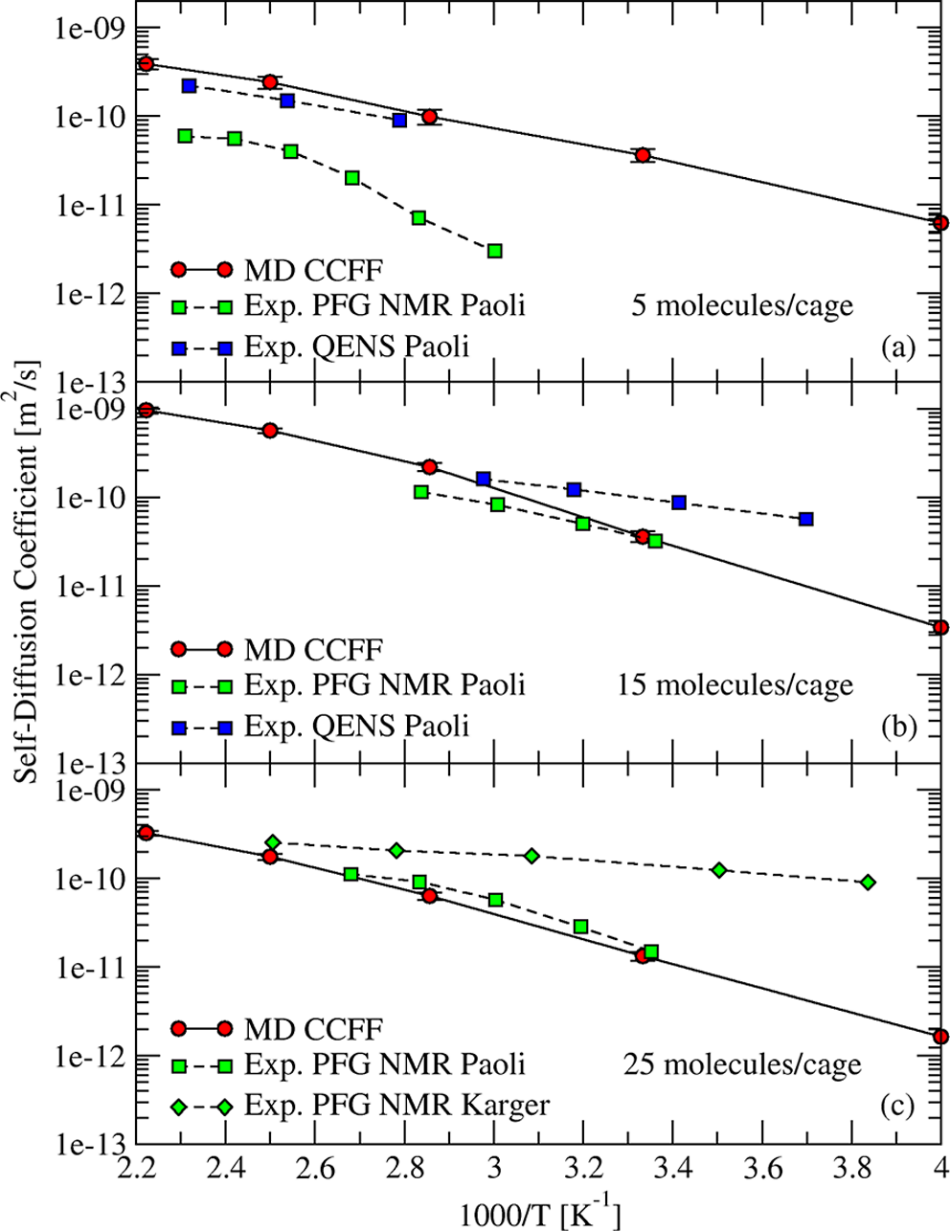
Temperature dependence of self-diffusion of H_2_O in 4A zeolite from PFG NMR and QENS experiments^93,94^ (squares and dashed curves) and simulations (circles and solid curves) at three different loadings.

Bourdin et al.^103^ and Karger^104^ reported the loading dependence of water diffusivity in NaX at 293 K using PFG NMR measurements (see Figure 14). The former work tested the effect of several activation modes and two different crystal sizes on the measurements. All experimental data showed diffusivities that were almost independent of loading to values close to saturation. Our simulations are in agreement with this observation except for loadings lower than 10 molecules/cage. Below this limit, difficulties in controlling loading and related uncertainties make PFG NMR measurements challenging.

**Figure 14:**
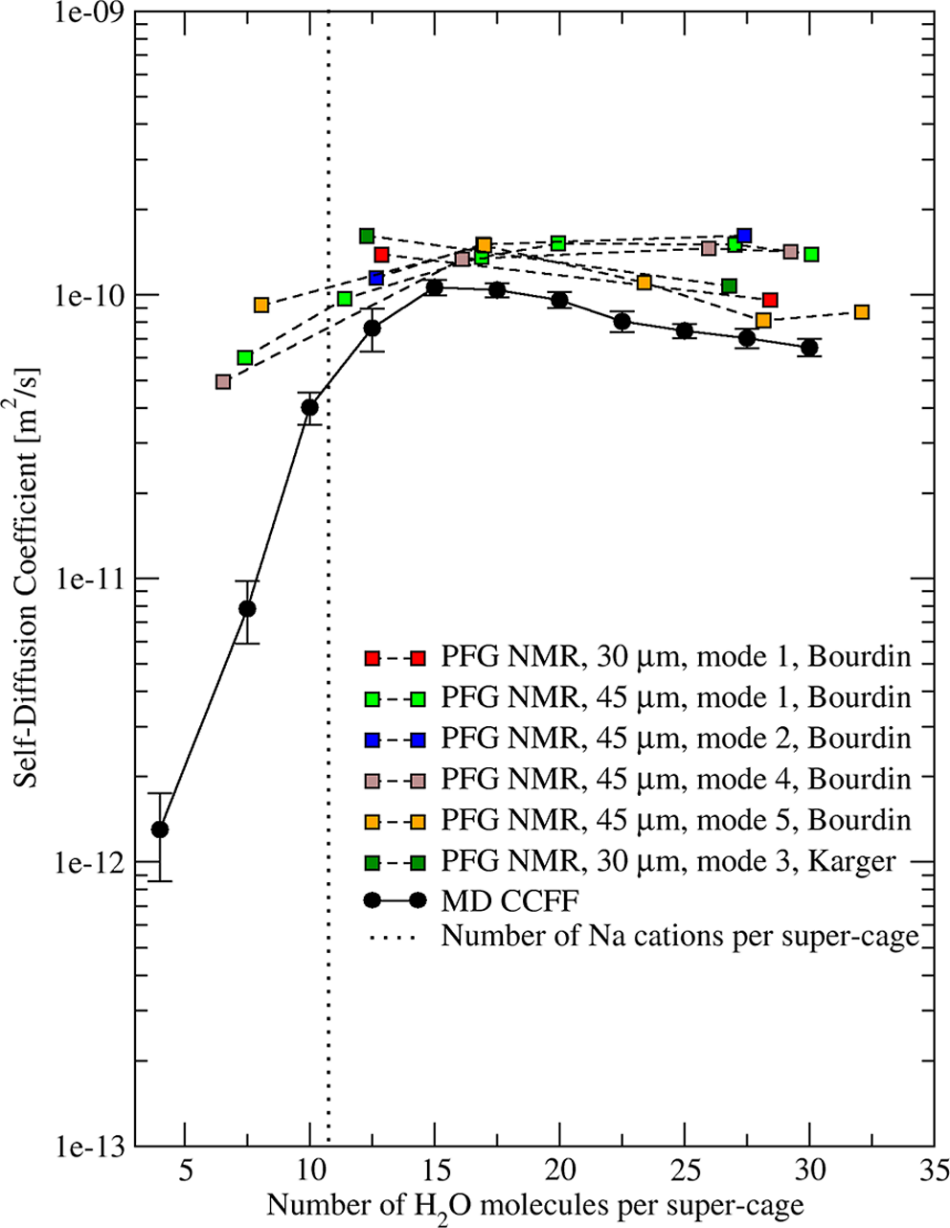
Simulated (circles and solid curve) and experimental^103,104^ (squares and dashed curves) loading dependence of H_2_O diffusion in NaX zeolite at 293 K. Five different modes were used to activate experimental samples. Dotted vertical line represents the number of Na cations per supercage.

For water self-diffusivity in 5A zeolite, the exact chemical composition of the samples was not reported, so we tested several experimental structures featuring two degrees of Na exchange, 67 and 83%, as shown in Figure 15a,b, respectively. For 5A structures with 67% exchange, the computed activation energy of water at a low loading of 5 molecules per cage was between 22 and 25 kJ/mol compared to 19 and 20 kJ/mol from PFG NMR and QENS experiments,^93,94^ respectively. For 5A structures with a higher degree of Na exchange (83%), the predicted activation energy was ∼28 kJ/mol, higher than experimental values derived from data shown in Figure 15b (12 and 19 kJ/mol using PFG NMR and QENS, respectively). This difference between simulated and experimental values may be due to lack of information about the exact chemical composition of commercial samples, difficulty in equilibrating low water loadings in 5A zeolite,^7,8^ or the sensitivity of Ca-rich zeolites to dehydration temperature.^105,106^

**Figure 15:**
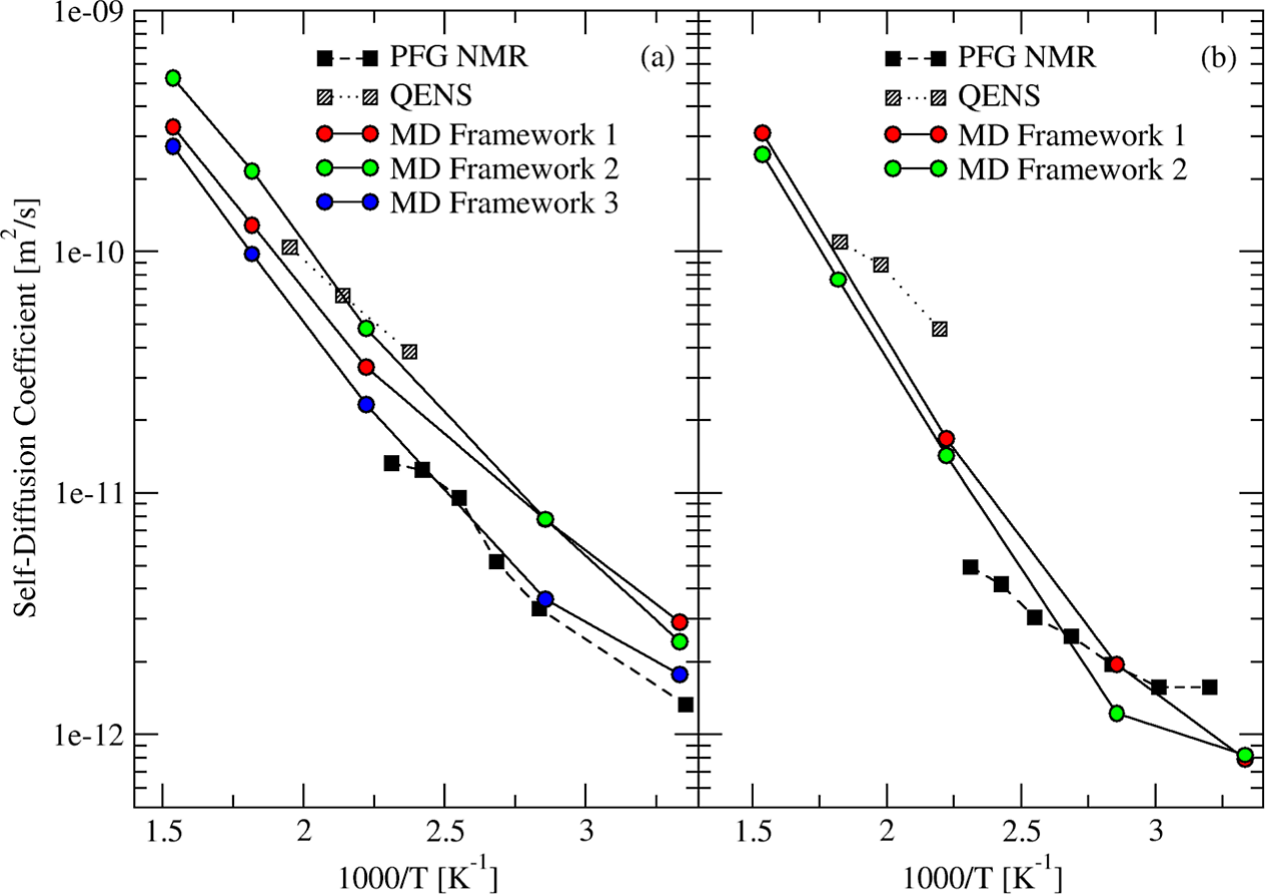
Simulated (circles and solid curves) and experimental (squares and dashed curves)^93,94^ temperature dependence of self-diffusion of H_2_O in 5A zeolite at loading of 5 molecules per cage. Different experimental frameworks^55–60^ of 5A zeolite with (a) 0.67 and (b) 0.83 Ca/Na ratios were used in simulations.

## 4 Conclusions

Water vapor is ubiquitous in separation processes and porous materials. The strong interactions between water and cations of aluminosilicates lead to relatively high heats of adsorption and the capacity of water in zeolites, which may affect the regeneration performance of these materials in a wide range of situations where exposure to humid streams is expected. To better understand and accurately predict water adsorption properties in cationic zeolites, we developed a force field between water and zeolites of monovalent (Li, Na, K, Rb, and Cs), divalent (Mg, Ca, Sr, and Ba) cations, and protons. The force field is transferable between different zeolite topologies and covers systems with different Si/Al ratios from fully exchanged (Si/Al = 1) to pure-silica (Si/Al = ∞) forms. The force field was fitted to highly accurate DFT/CC energies using training sets that include both energetically favorable and unfavorable states. This approach makes it possible to achieve high predictive accuracy for both the adsorption and diffusion properties of water in cationic zeolites.

To test predictions using the CCFF, we summarized available experimental data of water adsorption in widely used molecular sieves, such as 3A, 4A, and X zeolites. Our predictions showed very good agreement with experiments and helped explain the variation in some experimental data due to the presence of binders and the use of insufficiently strong regeneration temperatures. The transferability of CCFF enabled us to explore the correlation between the size of monovalent cations and water adsorption capacity and the effect of partial exchange in MNaX mixed systems (M = Li, K, Rb, and Cs). To demonstrate the capability of CCFF to predict both equilibrium adsorption properties and molecular diffusivity, we examined the temperature and loading dependence of water self-diffusivity in 4A, NaX, and 5A. Our results showed good agreement with experimental data using PFG NMR and QENS measurements.

To the best of our knowledge, CCFF is the first force field that enables the simulation of both water adsorption and diffusion in zeolites of monovalent and divalent cations with quantitative accuracy. The force field we have reported for adsorbed water can be combined with our previous versions of CCFF^13,14^ for other small molecules in pure-silica and zeolites with monovalent cations to explore the impacts of water coadsorption on the properties of these other molecules in zeolites.

## Supplementary Material





## Notes

Numerical data (isotherms, heats of adsorption, and self-diffusion coefficients) (XLSX)
CCFF parameters, fitting, additional validation of cation–framework interactions, and additional adsorption validation (PDF)

## References

[1] Corma A. (2003). State of the
art and future challenges of zeolites as catalysts. J. Catal..

[2] Auerbach, S. M. ; Carrado, K. A. ; Dutta, P. K. Handbook of Zeolite Science and Technology, 1st ed.; CRC Press, 2003.

[3] Tatlier M., Atalay-Oral C., Bayrak A., Maras T., Erdem A. (2022). Impact of
ion exchange on zeolite hydrophilicity/hydrophobicity monitored by
water capacity using thermal analysis. Thermochim.
Acta.

[4] Nascimento B. O., dos Santos B. F., Maia D. A. S., de Melo D. C., Vilarrasa-Garcia E., Torres A. E. B., Bastos-Neto M., Azevedo D. C. S. (2021). Water adsorption
in fresh and thermally aged zeolites: equilibrium and kinetics. Adsorption.

[5] Wang Y., Ivashko A., Carstensen B., Cundy S., Huang Q. L. (2023). Acid-resistant
zeolite RHO for deep dehydration. Sep. Purif.
Technol..

[6] Santos K. M. C., Menezes T. R., Oliveira M. R., Silva T. S. L., Santos K. S., Barros V. A., Melo D. C., Ramos A. L., Santana C. C., Franceschi E. (2021). Natural gas dehydration by adsorption using
MOFs and silicas: A review. Sep. Purif. Technol..

[7] Wang Y. (2020). Measurements
and Modeling of Water Adsorption Isotherms of Zeolite Linde-Type A
Crystals. Ind. Eng. Chem. Res..

[8] Wang Y., Levan M. D. (2009). Adsorption Equilibrium
of Carbon Dioxide and Water
Vapor on Zeolites 5A and 13X and Silica Gel: Pure Components. J. Chem. Eng. Data.

[9] Fang H., Kamakoti P., Zang J., Cundy S., Paur C., Ravikovitch P. I., Sholl D. S. (2012). Prediction of CO_2_ Adsorption
Properties in Zeolites Using Force Fields Derived from Periodic Dispersion-Corrected
DFT Calculations. J. Phys. Chem. C.

[10] Fang H. J., Kamakoti P., Ravikovitch P. I., Aronson M., Paur C., Sholl D. S. (2013). First principles
derived, transferable force fields
for CO_2_ adsorption in Na-exchanged cationic zeolites. Phys. Chem. Chem. Phys..

[11] Fang H., Kulkarni A., Kamakoti P., Awati R., Ravikovitch P. I., Sholl D. S. (2016). Identification of high-CO_2_-capacity cationic
zeolites by accurate computational screening. Chem. Mater..

[12] Fang H. J., Awati R., Boulfelfel S. E., Ravikovitch P. I., Sholl D. S. (2018). First-Principles-Derived Force Fields for CH_4_ Adsorption and Diffusion in Siliceous Zeolites. J. Phys. Chem. C.

[13] Findley J. M., Boulfelfel S. E., Fang H. J., Muraro G., Ravikovitch P. I., Sholl D. S. (2021). A Transferable Force Field for Predicting
Adsorption
and Diffusion of Hydrocarbons and Small Molecules in Silica Zeolites
with Coupled-Cluster Accuracy. J. Phys. Chem.
C.

[14] Boulfelfel S. E., Findley J. M., Fang H. J., Daou A. S. S., Ravikovitch P. I., Sholl D. S. (2021). A Transferable Force
Field for Predicting Adsorption
and Diffusion of Small Molecules in Alkali Metal Exchanged Zeolites
with Coupled Cluster Accuracy. J. Phys. Chem.
C.

[15] Fang H. J., Findley J., Muraro G., Ravikovitch P. I., Sholl D. S. (2020). A Strong Test of Atomically Detailed
Models of Molecular
Adsorption in Zeolites Using Multilaboratory Experimental Data for
CO_2_ Adsorption in Ammonium ZSM-5. J. Phys. Chem. Lett..

[16] Calero S., Dubbeldam D., Krishna R., Smit B., Vlugt T. J. H., Denayer J. F. M., Martens J. A., Maesen T. L. M. (2004). Understanding
the role of sodium during adsorption: A force field for alkanes in
sodium-exchanged faujasites. J. Am. Chem. Soc..

[17] Garcia-Perez E., Dubbeldam D., Maesen T. L. M., Calero S. (2006). Influence of cation
Na/Ca ratio on adsorption in LTA 5A: A systematic molecular simulation
study of alkane chain length. J. Phys. Chem.
B.

[18] Daou A. S. S., Findley J. M., Fang H. J., Boulfelfel S. E., Ravikovitch P. I., Sholl D. S. (2021). Quantifying Impact
of Intrinsic Flexibility
on Molecular Adsorption in Zeolites. J. Phys.
Chem. C.

[19] Berendsen H. J. C., Grigera J. R., Straatsma T. P. (1987). The Missing Term in Effective Pair
Potentials. J. Phys. Chem..

[20] Kresse G., Hafner J. (1994). Ab-Initio Molecular-Dynamics Simulation
of the Liquid-Metal
Amorphous-Semiconductor Transition in Germanium. Phys. Rev. B:Condens. Matter Mater. Phys..

[21] Kresse G., Furthmuller J. (1996). Efficient
iterative schemes for ab initio total-energy
calculations using a plane-wave basis set. Phys.
Rev. B:Condens. Matter Mater. Phys..

[22] Kresse G., Joubert D. (1999). From ultrasoft pseudopotentials
to the projector augmented-wave
method. Phys. Rev. B:Condens. Matter Mater.
Phys..

[23] Blochl P. E. (1994). Projector
Augmented-Wave Method. Phys. Rev. B:Condens.
Matter Mater. Phys..

[24] Grimme S. (2006). Semiempirical
GGA-type density functional constructed with a long-range dispersion
correction. J. Comput. Chem..

[25] Bludsky O., Rubes M., Soldan P., Nachtigall P. (2008). Investigation
of the benzene-dimer potential energy surface: DFT/CCSD­(T) correction
scheme. J. Chem. Phys..

[26] Nachtigall P., Delgado M. R., Nachtigallova D., Areán C. O. (2012). The nature
of cationic adsorption sites in alkaline zeolites—single, dual
and multiple cation sites. Phys. Chem. Chem.
Phys..

[27] Pulido A., Delgado M., Bludský O., Rubeš M., Nachtigall P., Areán C. O. (2009). Combined
DFT/CC and IR spectroscopic
studies on carbon dioxide adsorption on the zeolite H-FER. Energy Environ. Sci..

[28] Arean C. O., Palomino G. T., Carayol M. R. L., Pulido A., Rubes M., Bludsky O., Nachtigall P. (2009). Hydrogen adsorption on the zeolite
Ca-A: DFT and FT-IR investigation. Chem. Phys.
Lett..

[29] Manz T. A., Limas N. G. (2016). Introducing DDEC6
atomic population analysis: part
1. Charge partitioning theory and methodology. RSC Adv..

[30] Limas N. G., Manz T. A. (2016). Introducing DDEC6
atomic population analysis: part
2. Computed results for a wide range of periodic and nonperiodic materials. RSC Adv..

[31] Cygan R. T., Liang J. J., Kalinichev A. G. (2004). Molecular models of hydroxide, oxyhydroxide,
and clay phases and the development of a general force field. J. Phys. Chem. B.

[32] Jaramillo E., Auerbach S. M. (1999). New force field for Na cations in faujasite-type zeolites. J. Phys. Chem. B.

[33] Fang H. J., Daou A. S. S., Boulfelfel S. E., Findley J. M., Ravikovitch P. I., Sholl D. S. (2023). Computational Screening of Cationic Zeolites for-Butane/Methane
Separations Using Quantitatively Accurate First-Principles-Derived
Force Fields. J. Phys. Chem. C.

[34] Fang H., Kamakoti P., Ravikovitch P. I., Aronson M., Paur C., Sholl D. S. (2013). First principles
derived, transferable force fields
for CO_2_ adsorption in Na-exchanged cationic zeolites. Phys. Chem. Chem. Phys..

[35] Pirngruber G., Raybaud P., Belmabkhout Y., Čejka J., Zukal A. (2010). The role of the extra-framework cations in the adsorption of CO_2_ on faujasite Y. Phys. Chem. Chem. Phys..

[36] Yang X., Epiepang F. E., Liu Y., Yang R. T. (2018). Heats of adsorption
on mixed-cation LiNa-LSX: Estimating SIII site occupancy by Li. Chem. Eng. Sci..

[37] Dubbeldam D., Calero S., Ellis D. E., Snurr R. Q. (2016). RASPA: molecular
simulation software for adsorption and diffusion in flexible nanoporous
materials. Mol. Simul..

[38] Beauvais C., Guerrault X., Coudert F.-X., Boutin A., Fuchs A. H. (2004). Distribution
of sodium cations in faujasite-type zeolite: a canonical parallel
tempering simulation study. J. Phys. Chem. B.

[39] Findley J. M., Ravikovitch P. I., Sholl D. S. (2018). The effect of aluminum short-range
ordering on carbon dioxide adsorption in zeolites. J. Phys. Chem. C.

[40] Agrawal M., Sholl D. S. (2019). Effects of Intrinsic Flexibility
on Adsorption Properties
of Metal-Organic Frameworks at Dilute and Nondilute Loadings. ACS Appl. Mater. Interfaces.

[41] Witman M., Ling S., Jawahery S., Boyd P. G., Haranczyk M., Slater B., Smit B. (2017). The influence of intrinsic
framework
flexibility on adsorption in nanoporous materials. J. Am. Chem. Soc..

[42] Vlugt T. J., Schenk M. (2002). Influence of framework flexibility on the adsorption
properties of hydrocarbons in the zeolite silicalite. J. Phys. Chem. B.

[43] García-Sánchez A., Dubbeldam D., Calero S. (2010). Modeling adsorption and self-diffusion
of methane in LTA zeolites: the influence of framework flexibility. J. Phys. Chem. C.

[44] Fang H., Findley J., Muraro G., Ravikovitch P. I., Sholl D. S. (2020). A Strong Test of Atomically-detailed Models of Molecular
Adsorption in Zeolites Using Multi-laboratory Experimental Data for
CO_2_ Adsorption in Ammonium ZSM-5. J. Phys. Chem. Lett..

[45] Daou A. S. S., Findley J. M., Fang H., Boulfelfel S. E., Ravikovitch P. I., Sholl D. S. (2021). Quantifying Impact
of Intrinsic Flexibility
on Molecular Adsorption in Zeolites. J. Phys.
Chem. C.

[46] Plimpton S. (1995). Fast Parallel
Algorithms for Short-Range Molecular-Dynamics. J. Comput. Phys..

[47] Frenkel, D. ; Smit, B. Chapter 6 - Molecular Dynamics in Various Ensembles. In Understanding Molecular Simulation, 2nd ed.; Academic Press, 2002; pp 139–163.

[48] Frenkel, D. ; Smit, B. Appendix E-Integration Schemes. In Understanding Molecular Simulation, 2nd ed.; Academic Press, 2002; pp 533–544.

[49] Frenkel, D. ; Smit, B. Chapter 12 - Long-Range Interactions. In Understanding Molecular Simulation, 2nd ed.; Academic Press, 2002; pp 291–320.

[50] Sholl D. S. (2006). Understanding
macroscopic diffusion of adsorbed molecules in crystalline nanoporous
materials via atomistic simulations. Acc. Chem.
Res..

[51] Rakhmatkarieva F., Davlatova O., Kokhkharov M., Xudoyberganov M., Ergashev O., Abdurakhmonov E., Abdulkhaev T. (2023). Mechanism
of H_2_O Vapor Adsorption in A Type Zeolites: A Model Based
on Adsorption Calorimetry. E3S Web Conf..

[52] van
Kampen J., Boon J., van Sint Annaland M. (2021). Steam adsorption
on molecular sieve 3A for sorption enhanced reaction processes. Adsorption.

[53] Simo M., Sivashanmugam S., Brown C. J., Hlavacek V. (2009). Adsorption/Desorption
of Water and Ethanol on 3A Zeolite in Near-Adiabatic Fixed Bed. Ind. Eng. Chem. Res..

[54] Zhang A. D., Zeng W. X., Niemczyk T. M., Keenan M. R., Haaland D. M. (2005). Multivariate
analysis of infrared spectra for monitoring and understanding the
kinetics and mechanisms of adsorption processes. Appl. Spectrosc..

[55] Luhrs H., Derr J., Fischer R. X. (2012). K and Ca exchange behavior of zeolite
A. Microporous Mesoporous Mater..

[56] Broussard L., Shoemaker D. P. (1960). The Structures
of Synthetic Molecular Sieves. J. Am. Chem.
Soc..

[57] Seff K., Shoemaker D. P. (1967). Structures
of Zeolite Sorption Complexes. I. Structures
of Dehydrated Zeolite 5A and Its Iodine Sorption Complex. Acta Crystallogr..

[58] Siegel H., Schollner R., Staudte B., Vandun J. J., Mortier W. J. (1987). X-Ray Structural
Investigations on Hydrothermally Treated (Ca_4_, Na_4_)-A Zeolites. Zeolites.

[59] Jang S.-B., Song S. H., Kim Y. (1995). Crystal structures
of bromine sorption
complexes of Ca^2+^-exchanged zeolite A. Bull. Korean Chem. Soc..

[60] Adams J. M., Haselden D. A. (1984). The Structure of Dehydrated Zeolite
5A (Si/Al = 1.02)
by Neutron Profile Refinement. J. Solid State
Chem..

[61] AbdulKareem F. A., Shariff A. M., Ullah S., See T. L., Keong L. K., Mellon N. (2018). Adsorption performance
of 5A molecular sieve zeolite
in water vapor-binary gas environment: Experimental and modeling evaluation. J. Ind. Eng. Chem..

[62] Azhagapillai P., Khaleel M., Zoghieb F., Luckachan G., Jacob L., Reinalda D. (2022). Water Vapor Adsorption
Capacity Loss
of Molecular Sieves 4A, 5A, and 13X Resulting from Methanol and Heptane
Exposure. ACS Omega.

[63] Volavšek J., Pliekhov O., Pliekhova O., Mali G., Zabukovec
Logar N. (2022). Study of Water Adsorption on EDTA-Modified LTA Zeolites. Nanomaterials.

[64] Zagorac D., Mueller H., Ruehl S., Zagorac J., Rehme S. (2019). Recent developments
in the Inorganic Crystal Structure Database: theoretical crystal structure
data and related features. J. Appl. Crystallogr..

[65] Ahn H., Lee C. H. (2004). Effects of capillary
condensation on adsorption and
thermal desorption dynamics of water in zeolite 13X and layered beds. Chem. Eng. Sci..

[66] Chen D., Hu X., Shi L., Cui Q., Wang H. Y., Yao H. Q. (2012). Synthesis
and characterization of zeolite X from lithium slag. Appl. Clay Sci..

[67] Gopal R., Hollebone B. R., Langford C. H., Shigeishi R. A. (1982). The Rates
of Solar-Energy Storage and Retrieval in a Zeolite-Water System. Sol. Energy.

[68] Mette B., Kerskes H., Druck H., Muller-Steinhagen H. (2014). Experimental
and numerical investigations on the water vapor adsorption isotherms
and kinetics of binderless zeolite 13X. Int.
J. Heat Mass Tran..

[69] Ryu Y. K., Lee S. J., Kim J. W., Leef C. H. (2001). Adsorption equilibrium
and kinetics of H_2_O on zeolite 13X. Korean J. Chem. Eng..

[70] Semprini S., Lehmann C., Beckert S., Kolditz O., Glaser R., Kerskes H., Nagel T. (2017). Numerical modelling of water sorption
isotherms of zeolite 13XBF based on sparse experimental data sets
for heat storage applications. Energy Convers.
Manag..

[71] Son K. N., Richardson T. M. J., Cmarik G. E. (2019). Equilibrium Adsorption Isotherms
for H_2_O on Zeolite 13X. J. Chem.
Eng. Data.

[72] Wynnyk K. G., Hojjati B., Marriott R. A. (2018). High-Pressure Sour Gas and Water
Adsorption on Zeolite 13X. Ind. Eng. Chem. Res..

[73] Chuikina V. K., Kiselev A. V., Mineyeva L. V., Muttik G. G. (1976). Heats of Adsorption
of Water-Vapor on NaX and KNaX Zeolites at Different Temperatures. J. Chem. Soc., Faraday Trans. 1.

[74] Zhu R. Q., Han B. Q., Lin M. Z., Yu Y. Z. (1992). Experimental Investigation
on an Adsorption System for Producing Chilled Water. Int. J. Refrig..

[75] Kim J. H., Lee C. H., Kim W. S., Lee J. S., Kim J. T., Suh J. K., Lee J. M. (2003). Adsorption equilibria
of water vapor
on alumina, zeolite 13X, and a zeolite X/activated carbon composite. J. Chem. Eng. Data.

[76] Li G., Xiao P., Webley P. A., Zhang J., Singh R. (2009). Competition
of CO_2_/H_2_O in Adsorption Based CO_2_ Capture. Energy Procedia.

[77] Ferreira D., Magalhaes R., Taveira P., Mendes A. (2011). Effective Adsorption
Equilibrium Isotherms and Breakthroughs of Water Vapor and Carbon
Dioxide on Different Adsorbents. Ind. Eng. Chem.
Res..

[78] Hefti M., Marx D., Joss L., Mazzotti M. (2015). Adsorption equilibrium
of binary mixtures of carbon dioxide and nitrogen on zeolites ZSM-5
and 13X. Microporous Mesoporous Mater..

[79] Kim K. M., Oh H. T., Lim S. J., Ho K., Park Y., Lee C. H. (2016). Adsorption Equilibria of Water Vapor
on Zeolite 3A,
Zeolite 13X, and Dealuminated Y Zeolite. J.
Chem. Eng. Data.

[80] Lehmann C., Beckert S., Nonnen T., Glaser R., Kolditz O., Nagel T. (2017). Water loading lift and heat storage
density prediction of adsorption
heat storage systems using Dubinin-Polanyi theory-Comparison with
experimental results. Appl. Energy.

[81] Son, K. N. Improved Prediction of Adsorption-Based Life Support for Deep Space Exploration; Purdue University Graduate School, 2019. https://hammer.purdue.edu/articles/thesis/Improved_Prediction_of_Adsorption-Based_Life_Support_for_Deep_Space_Exploration/7423451.

[82] Schumann K., Unger B., Brandt A., Scheffler F. (2012). Investigation
on the pore structure of binderless zeolite 13X shapes. Microporous Mesoporous Mater..

[83] Brandani F., Ruthven D. M. (2004). The effect of water
on the adsorption of CO_2_ and C_3_H_8_ on type X zeolites. Ind. Eng. Chem. Res..

[84] Ammouli T., Paillaud J. L., Nouali H., Stephan R., Hanf M. C., Sonnet P., Deroche I. (2021). Insights into
Water Adsorption in
Potassium-Exchanged X-type Faujasite Zeolite: Molecular Simulation
and Experiment. J. Phys. Chem. C.

[85] Dzhigit O. M., Kiselev A. V., Mikos K. N., Muttik G. G., Rahmanova T. A. (1971). Heats of
Adsorption of Water Vapour on X-Zeolites Containing Li^+^, Na^+^, K^+^, Rb^+^ and Cs^+^ Cations. Trans. Faraday Soc..

[86] Avgul N. N., Bezus A. G., Dzhigit O. M. (1971). Heats of Adsorption on X-Type Zeolites
Containing Different Alkali Metal Cations. Adv.
Chem. Ser..

[87] Joshi U. D., Joshi P. N., Tamhankar S. S., Joshi V. P., Idage B. B., Joshi V. V., Shiralkar V. P. (2002). Influence
of the size of extraframework
monovalent cations in X-type zeolite on their thermal behavior. Thermochim. Acta.

[88] Fritsch F. N., Carlson R. E. (1980). Monotone Piecewise Cubic Interpolation. SIAM J. Numer. Anal..

[89] Morris B. (1968). Heats of Sorption
in Crystalline Linde-a Zeolite-Water Vapor System. J. Colloid Interface Sci..

[90] Dzhigit O. M., Kiselev A. V., Mikos K. N., Muttik G. G. (1964). Heat of
Adsorption
of Water Vapors on Zeolite of the Type of Na-Faujasite. Zh. Fiz. Khim..

[91] Dubinin M. M., Isirikyan A. A., Rakhmatkariev G. U., Serpinskii V. V. (1973). Differential
adsorption heats of water on powdered synthetic zeolite NaX. Bull. Acad. Sci. USSR.

[92] Dzhigit O. M., Kiselev A. V., Rachmanova T. A., Zhdanov S. P. (1979). Influence of Li^+^, Na^+^ and K^+^ Cation Concentrations in
X and Y Zeolites on Isotherms and Heats of Adsorption of Propane and
Water. J. Chem. Soc., Faraday Trans. 1.

[93] Paoli H., Methivier A., Jobic H., Krause C., Pfeifer H., Stallmach F., Karger J. (2002). Comparative QENS and
PFG NMR diffusion
studies of water in zeolite NaCaA. Microporous
Mesoporous Mater..

[94] Karger J., Pfeifer H., Rosemann M., Feokistova N. N., Zdanov S. P. (1989). Intracrystalline Self-Diffusion of Water and Short-Chain-Length
Paraffins in A-Type Zeolites. Zeolites.

[95] Jobic H., Méthivier A. (2005). Intracrystalline
diffusion in zeolites studied by neutron
scattering techniques. Oil Gas Sci. Technol..

[96] Valiullin R., Kärger J., Cho K., Choi M., Ryoo R. (2011). Dynamics of
water diffusion in mesoporous zeolites. Microporous
Mesoporous Mater..

[97] Kamitakahara W. A., Wada N. (2008). Neutron spectroscopy
of water dynamics in NaX and NaA zeolites. Phys.
Rev. E:Stat., Nonlinear, Soft Matter Phys..

[98] Jensen B., Kuznetsova T., Kvamme B., Olsen R. (2015). The impact
of electrostatics
in bulk Linde Type A zeolites. Microporous Mesoporous
Mater..

[99] Turgman-Cohen S., Araque J. C., Hoek E. M. V., Escobedo F. A. (2013). Molecular Dynamics
of Equilibrium and Pressure-Driven Transport Properties of Water through
LTA-Type Zeolites. Langmuir.

[100] Demontis P., Gulin-Gonzalez J., Jobic H., Masia M., Sale R., Suffritti G. B. (2008). Dynamical properties of confined
water nanoclusters: Simulation study of hydrated zeolite NaA: Structural
and vibrational properties. ACS Nano.

[101] Demontis P., Gulín-González J., Jobic H., Suffritti G. B. (2010). Diffusion of Water in Zeolites NaA
and NaCaA: A Molecular Dynamics Simulation Study. J. Phys. Chem. C.

[102] Kärger J., Avramovska M., Freude D., Haase J., Hwang S., Valiullin R. (2021). Pulsed field gradient NMR diffusion
measurement in nanoporous materials. Adsorption.

[103] Bourdin V., Germanus A., Grenier P., Karger J. (1996). Application
of the thermal frequency response method and of pulsed field gradient
NMR to study water diffusion in zeolite NaX. Adsorption.

[104] Karger J. (1971). Diffusion Study of Water on 13X,
4A and 5A Zeolites
Using Pulsed Field Gradient Technique. Z. Phys.
Chem..

[105] Deroy G., Vansant E. F., Hendrickx M. (1980). Sorption in
Partially Hydrated Naa and Caa Zeolites. J.
Chem. Soc., Faraday Trans. 1.

[106] Michelena J. A., Vansant E. F., Debievre P. (1977). Effect of Pretreatment
of Caa Zeolite on Adsorption of Co. Recl. Trav.
Chim. Pays-Bas.

