# Supplementary material for: A Transferable Force Field for Predicting Adsorption and Diffusion of Water in Cationic Zeolites with Coupled Cluster Accuracy
Source: ACS Phys Chem Au. 2025 Aug 8;5(5):533–48. doi: 10.1021/acsphyschemau.5c00038 (PMC12464758; doi:10.1021/acsphyschemau.5c00038)
Supplement: Supplementary file 2 [file pg5c00038_si_002.pdf]

SUPPORTING INFORMATION FOR

A Transferable Force Field for Predicting Adsorption and Diffusion of Water in Cationic Zeolites with Coupled Cluster Accuracy: Supporting Information

Salah Eddine Boulfelfel[1], Hanjun Fang[1], Alan S. S. Daou[1], Peter I. Ravikovitch[2†*], and David S. Sholl[3‡*]

[1]School of Chemical and Biomolecular Engineering, Georgia Institute of Technology, Atlanta, Georgia 30332-0100, United States

[2]ExxonMobil Technology and Engineering Company, 1545 Route 22 East, Annandale, New Jersey 08801, United States

[3]Oak Ridge National Laboratory, Oak Ridge, TN 37830, United States

† +1-908-730-2280, peter.ravikovitch@exxonmobil.com

‡ +1-865-341-1743, shollds@ornl.gov

Table of Contents

S1. ASSOCIATED CONTENT DESCRIPTION **3**

S1. CC CORRECTION TABLES **3**

S2. FORCE FIELD FITTING RESULTS **4**

S3. FORCE FIELD VALIDATION RESULTS **10**

S1. Associated content description

Numerical data of figures from the main text (Figs 1–7 isotherms, Figs 8–12 heats of adsorption, and Figs 13–15 self-diffusion coefficients) are tabulated in an Excel file.

S1. CC correction tables

Table S1: The DFT/CC correction functions ε_{ij} (in Hartree) as a function of atom-atom distances R_{ij} (in Å) for H_2O in silica zeolites; *i* stands for O and H atoms of H_2O and *j* stands for framework O and Si atoms. These values are taken from our previous work [Findley et al., J Phys Chem C 2021, 125, 8418-8429]. The correction functions between the atoms of H_2O and Si are updated here because the original ones were incorrectly presented.

R_{OO}	ε_{OO}	R_{HO}	ε_{HO}	R_{OSi}	ε_{OSi}	R_{HSi}	ε_{HSi}
1.800000	0.00374316	1.000000	0.00223892	2.044660	0.02097000	2.308870	0.00198000
2.000000	0.00151827	1.200000	0.00186688	2.177560	0.01774000	2.443220	0.00123000
2.400000	0.00062977	1.400000	0.00094841	2.320100	0.00429000	2.586080	0.00108000
2.600000	0.00067160	1.600000	0.00020084	2.470620	-0.00361000	2.736100	0.00102000
3.000000	0.00073427	1.800000	-0.00023578	2.627740	-0.00450000	2.892180	0.00083821
3.200000	0.00070863	2.000000	-0.00044216	2.790360	-0.00357000	3.053390	0.00065858
3.400000	0.00065514	2.200000	-0.00049604	2.957570	-0.00303000	3.218950	0.00052341
3.600000	0.00058673	2.400000	-0.00047452	3.128630	-0.00278000	3.388230	0.00043599
3.800000	0.00051348	2.600000	-0.00042650	3.302940	-0.00253000	3.560700	0.00038326
4.000000	0.00044199	2.800000	-0.00036870	3.480020	-0.00221000	3.735910	0.00034208
4.200000	0.00037602	3.000000	-0.00031425	3.659460	-0.00188000	3.913500	0.00030532
4.400000	0.00031732	3.200000	-0.00027042	3.840930	-0.00159000	4.093160	0.00027285
4.600000	0.00026636	3.400000	-0.00023544	4.024150	-0.00134000	4.274620	0.00024360
4.800000	0.00022287	3.600000	-0.00020553	4.208910	-0.00113000	4.457670	0.00021611
5.000000	0.00018620	3.800000	-0.00017857	4.395000	-0.00094941	4.642120	0.00018977
5.500000	0.00011910	4.000000	-0.00015387	4.582260	-0.00079812	4.827810	0.00016485
6.000000	0.00007721	4.200000	-0.00013146	4.770560	-0.00066954	5.485650	0.00009566
6.500000	0.00005098	4.400000	-0.00011145	4.959770	-0.00056078	5.961420	0.00006394
7.000000	0.00003434	4.600000	-0.00009392	5.149800	-0.00046935	6.440860	0.00004300
8.000000	0.00001651	4.800000	-0.00007881	5.340550	-0.00039291	7.894500	0.00001427
9.000000	0.00000852	5.000000	-0.00006596	5.531960	-0.00032927	8.872170	0.00000740
11.000000	0.00000270	5.500000	-0.00004224	6.012930	-0.00021350	10.839740	0.00000235
14.000000	0.00000066	6.000000	-0.00002733	6.496770	-0.00014076	13.808580	0.00000057
19.000000	0.00000011	6.500000	-0.00001799	6.982900	-0.00009460	18.778670	0.00000009
		7.000000	-0.00001207	7.470850	-0.00006482		
		8.000000	-0.00000576	8.450980	-0.00003217		
		9.000000	-0.00000296	9.435280	-0.00001705		
		11.000000	-0.00000093	11.412080	-0.00000562		
		14.000000	-0.00000023	14.389300	-0.00000144		
		19.000000	-0.00000004	19.366970	-0.00000025		

S2. Force field fitting results

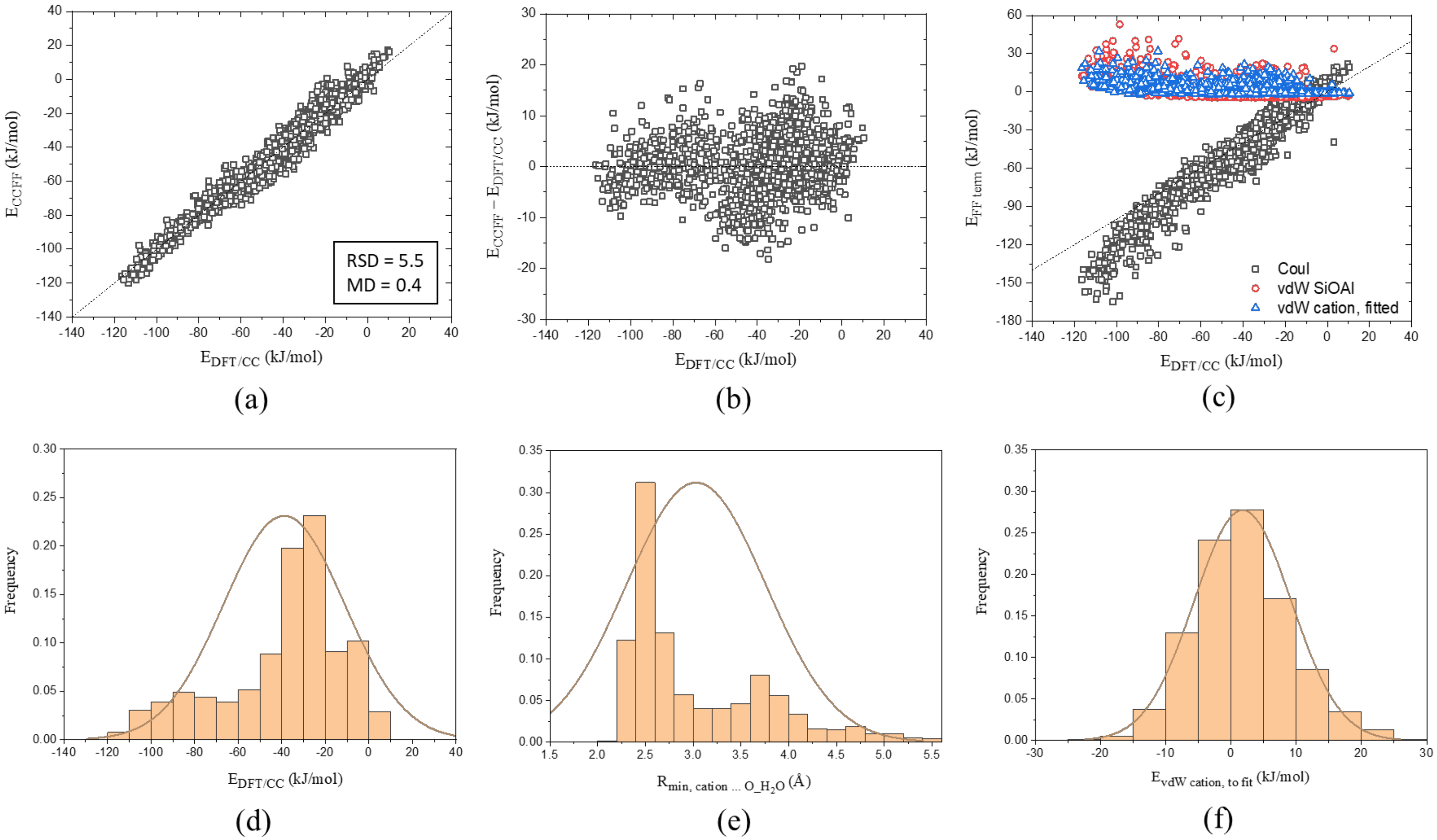

Figure S1. Force field fitting results for H_2O in Na-LTA (Si/Al = 1): (a) Comparison of the interaction energies for CCFF and DFT/CC, (b) the difference in interaction energies ($E_{CCFF} - E_{DFT/CC}$) as a function of $E_{DFT/CC}$, (c) van der Waals (vdW) and Coulomb (Coul) contributions from E_{CCFF} as a function of $E_{DFT/CC}$, distributions of (d) $E_{DFT/CC}$, (e) the minimum distance between cation and the O atom of H_2O, and (f) the vdW interaction of cation … H_2O that is to fit ($E_{vdW\ cation,\ to\ fit} = E_{DFT/CC} - E_{Coul} - E_{vdW\ SiOAl}$). RSD and MD in (a) represent residual standard deviation and mean deviation, respectively.

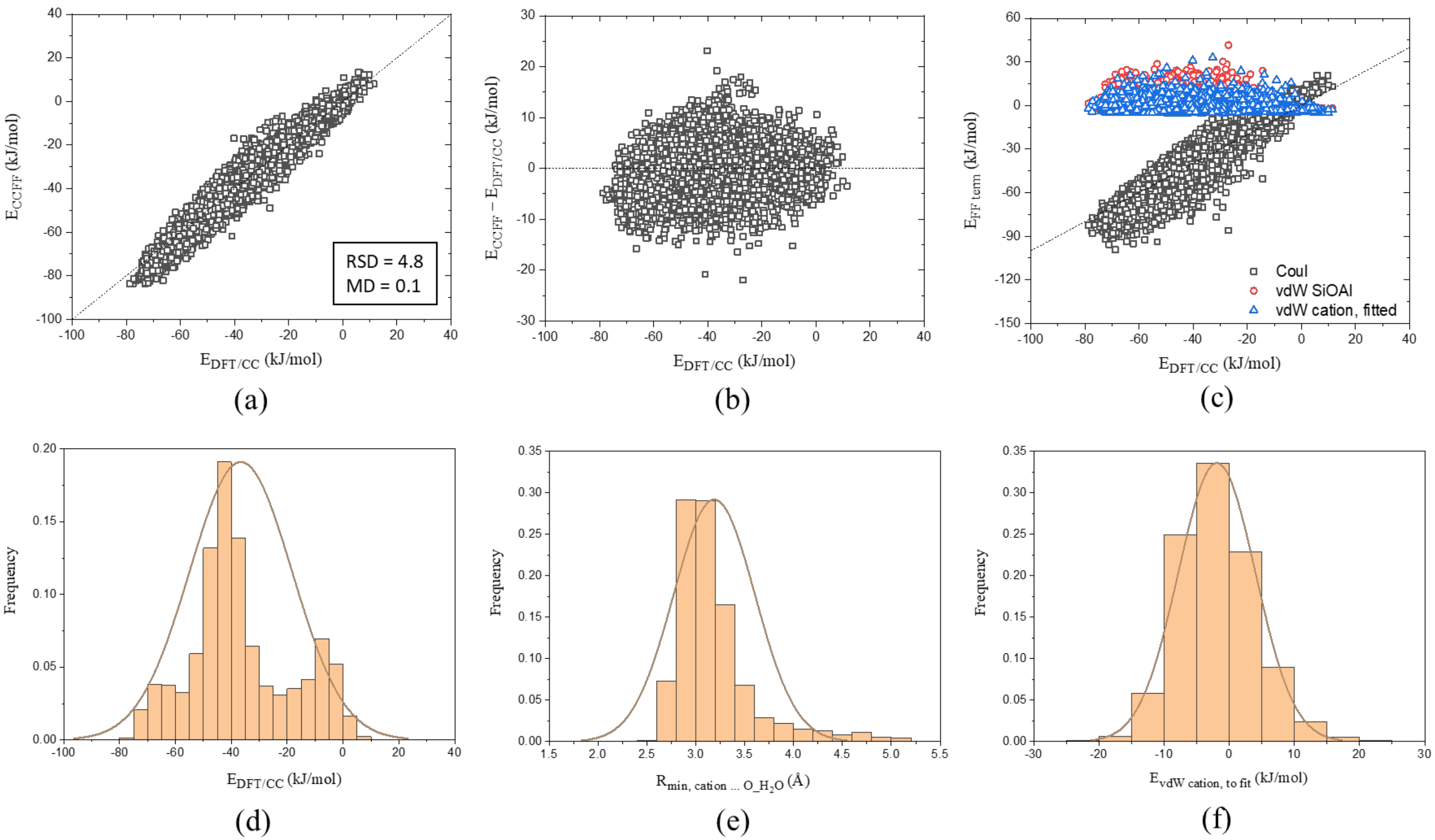

Figure S2. Force field fitting results for H_2O in K-LTA (Si/Al = 1): (a) Comparison of the interaction energies for CCFF and DFT/CC, (b) the difference in interaction energies ($E_{CCFF} - E_{DFT/CC}$) as a function of $E_{DFT/CC}$, (c) van der Waals (vdW) and Coulomb (Coul) contributions from E_{CCFF} as a function of $E_{DFT/CC}$, distributions of (d) $E_{DFT/CC}$, (e) the minimum distance between cation and the O atom of H_2O, and (f) the vdW interaction of cation … H_2O that is to fit ($E_{vdW\ cation,\ to\ fit} = E_{DFT/CC} - E_{Coul} - E_{vdW\ SiOAl}$). RSD and MD in (a) represent residual standard deviation and mean deviation, respectively.

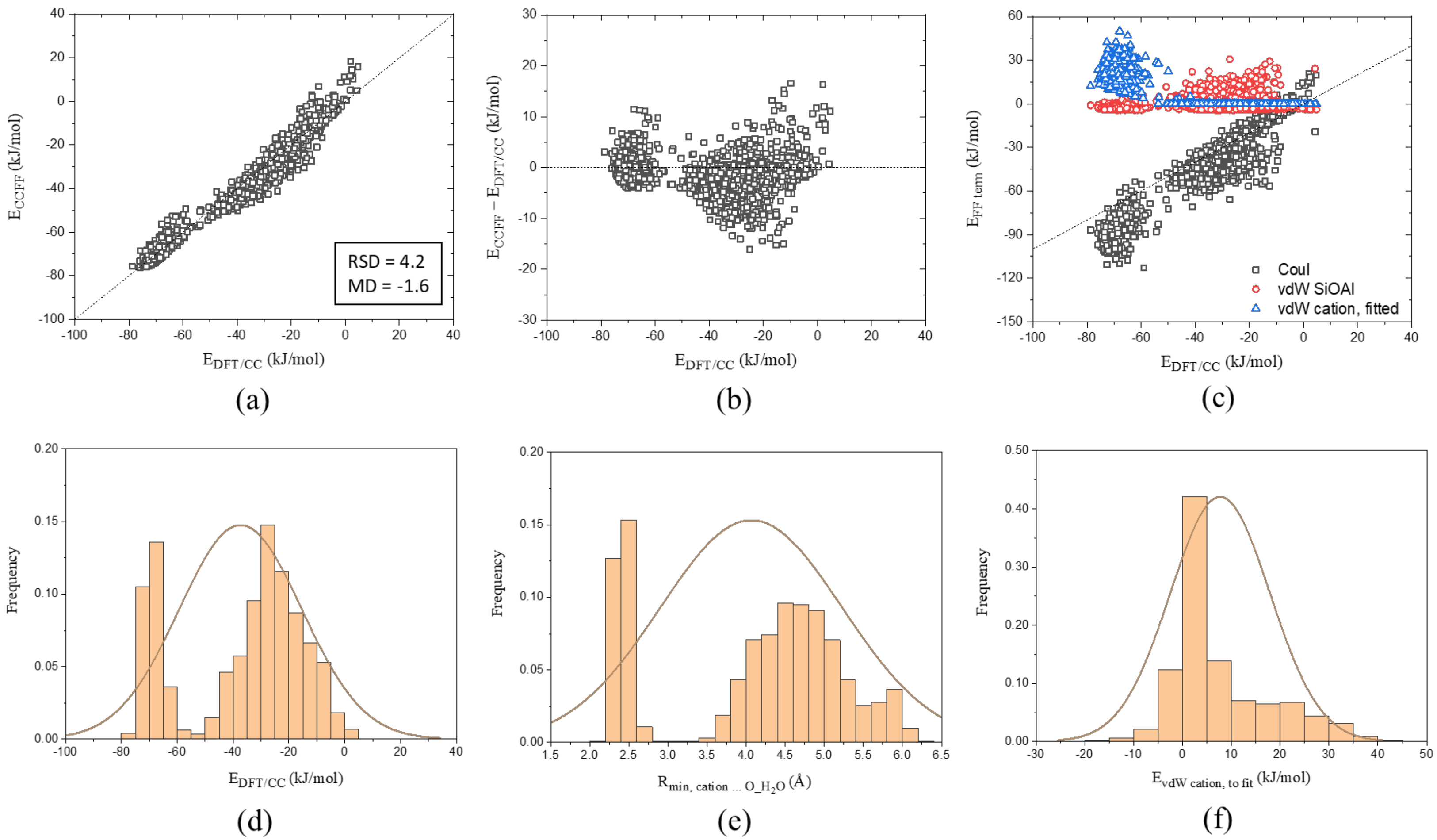

Figure S3. Force field fitting results for H_2O in Ca-LTA (Si/Al = 1): (a) Comparison of the interaction energies for CCFF and DFT/CC, (b) the difference in interaction energies ($E_{CCFF} - E_{DFT/CC}$) as a function of $E_{DFT/CC}$, (c) van der Waals (vdW) and Coulomb (Coul) contributions from E_{CCFF} as a function of $E_{DFT/CC}$, distributions of (d) $E_{DFT/CC}$, (e) the minimum distance between cation and the O atom of H_2O, and (f) the vdW interaction of cation … H_2O that is to fit ($E_{vdW\ cation,\ to\ fit} = E_{DFT/CC} - E_{Coul} - E_{vdW\ SiOAl}$). RSD and MD in (a) represent residual standard deviation and mean deviation, respectively.

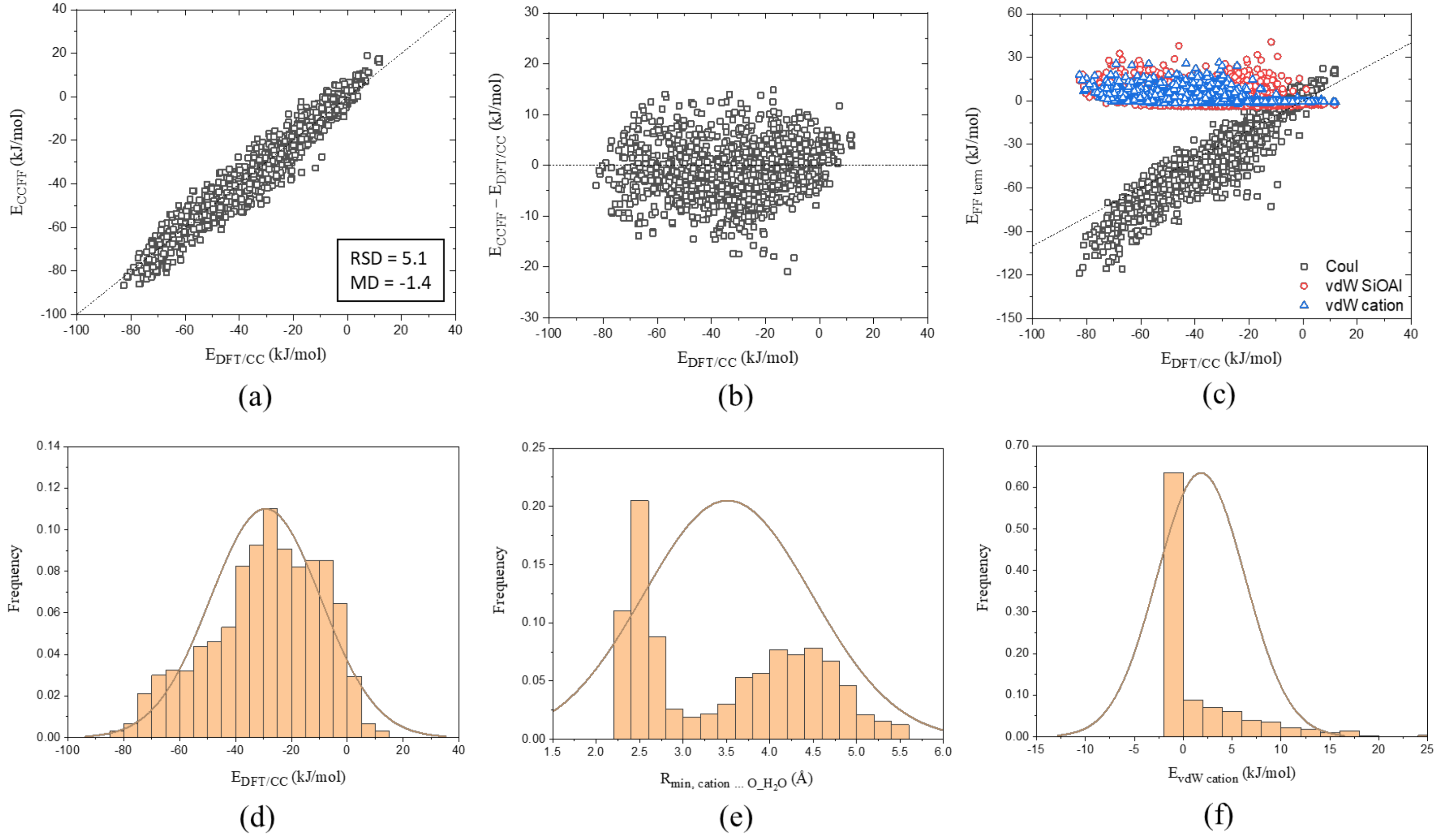

Figure S4. Force field transferability for H_2O in Na-FAU (Si/Al = 1): (a) Comparison of the interaction energies for CCFF and DFT/CC, (b) the difference in interaction energies ($E_{CCFF} - E_{DFT/CC}$) as a function of $E_{DFT/CC}$, (c) van der Waals (vdW) and Coulomb (Coul) contributions from E_{CCFF} as a function of $E_{DFT/CC}$, distributions of (d) $E_{DFT/CC}$, (e) the minimum distance between cation and the O atom of H_2O, and (f) the vdW interaction for cation with H_2O. RSD and MD in (a) represent residual standard deviation and mean deviation, respectively.

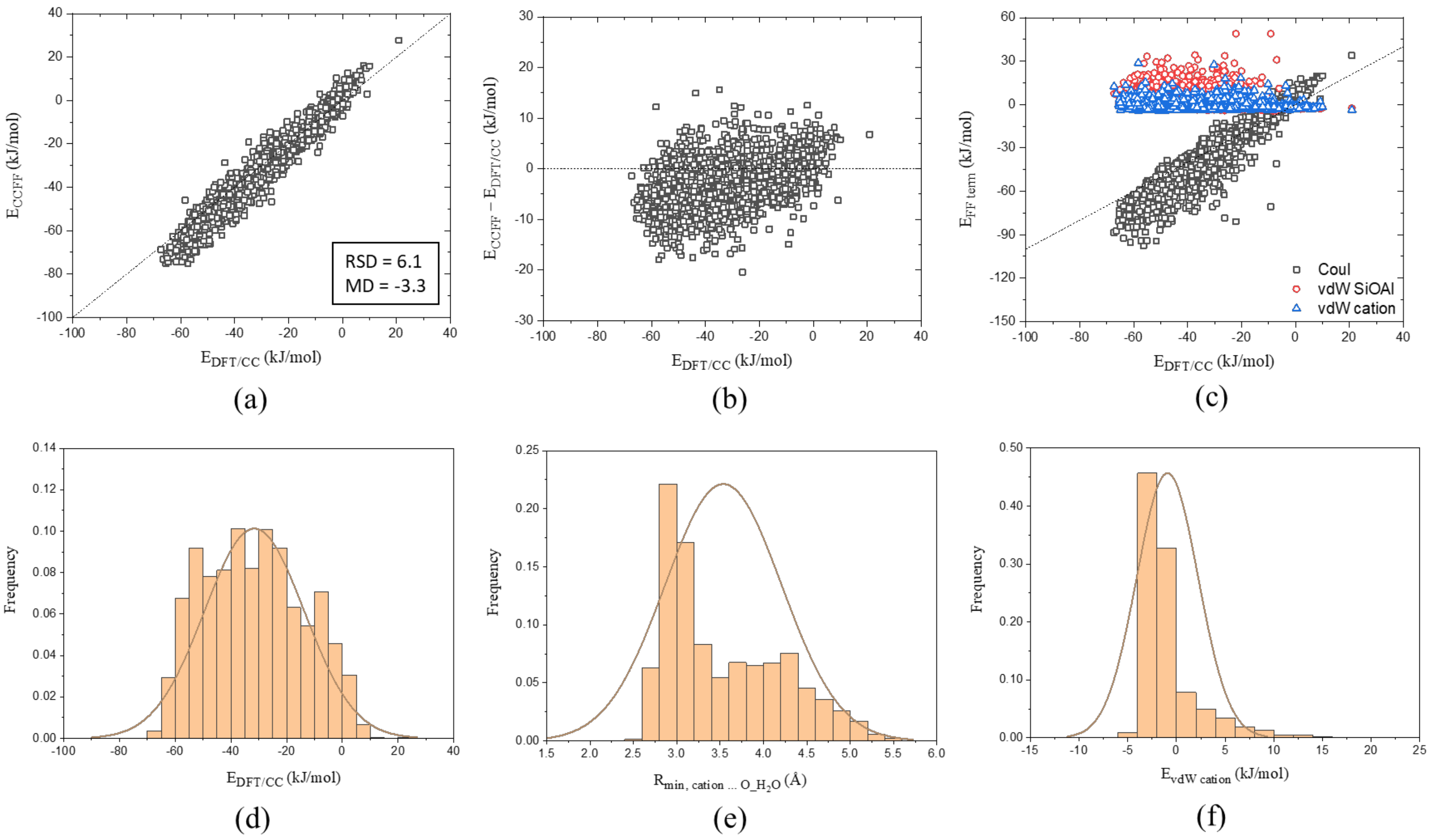

Figure S5. Force field transferability for H_2O in K-FAU (Si/Al = 1): (a) Comparison of the interaction energies for CCFF and DFT/CC, (b) the difference in interaction energies ($E_{CCFF} - E_{DFT/CC}$) as a function of $E_{DFT/CC}$, (c) van der Waals (vdW) and Coulomb (Coul) contributions from E_{CCFF} as a function of $E_{DFT/CC}$, distributions of (d) $E_{DFT/CC}$, (e) the minimum distance between cation and the O atom of H_2O, and (f) the vdW interaction for cation with H_2O. RSD and MD in (a) represent residual standard deviation and mean deviation, respectively.

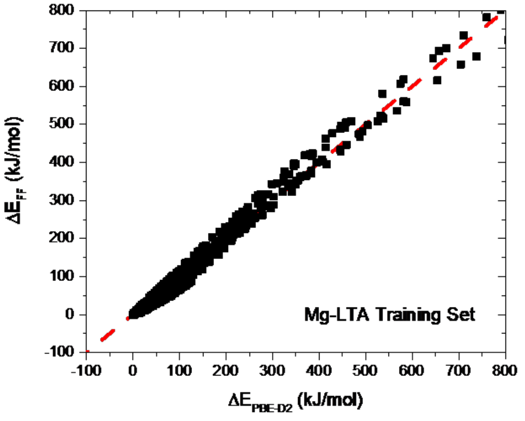

(a)

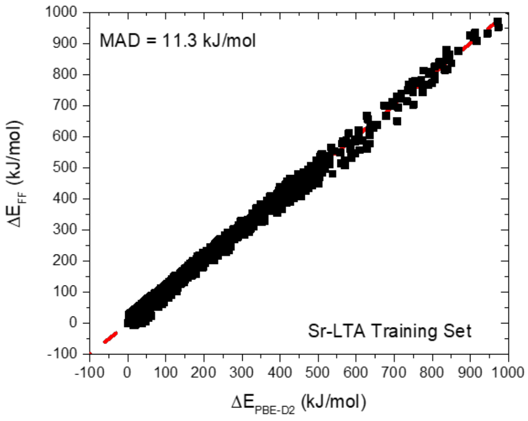

(b)

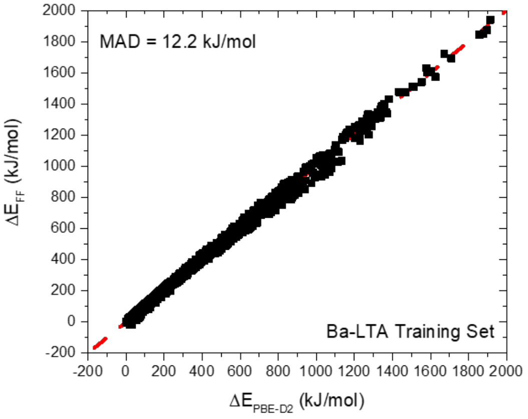

(c)

Figure S6. A comparison between PBE-D2 energies and CCFF energies that were fit to the PBE-D2 energies for the sets of configurations in (a) Mg-LTA, (b) Sr-LTA, and (c) Ba-LTA. Since correction functions between cation and zeolite framework atoms are not currently available in the DFT/CC method, the PBE-D2 method was used for these energy calculations.

S3. Force field validation results

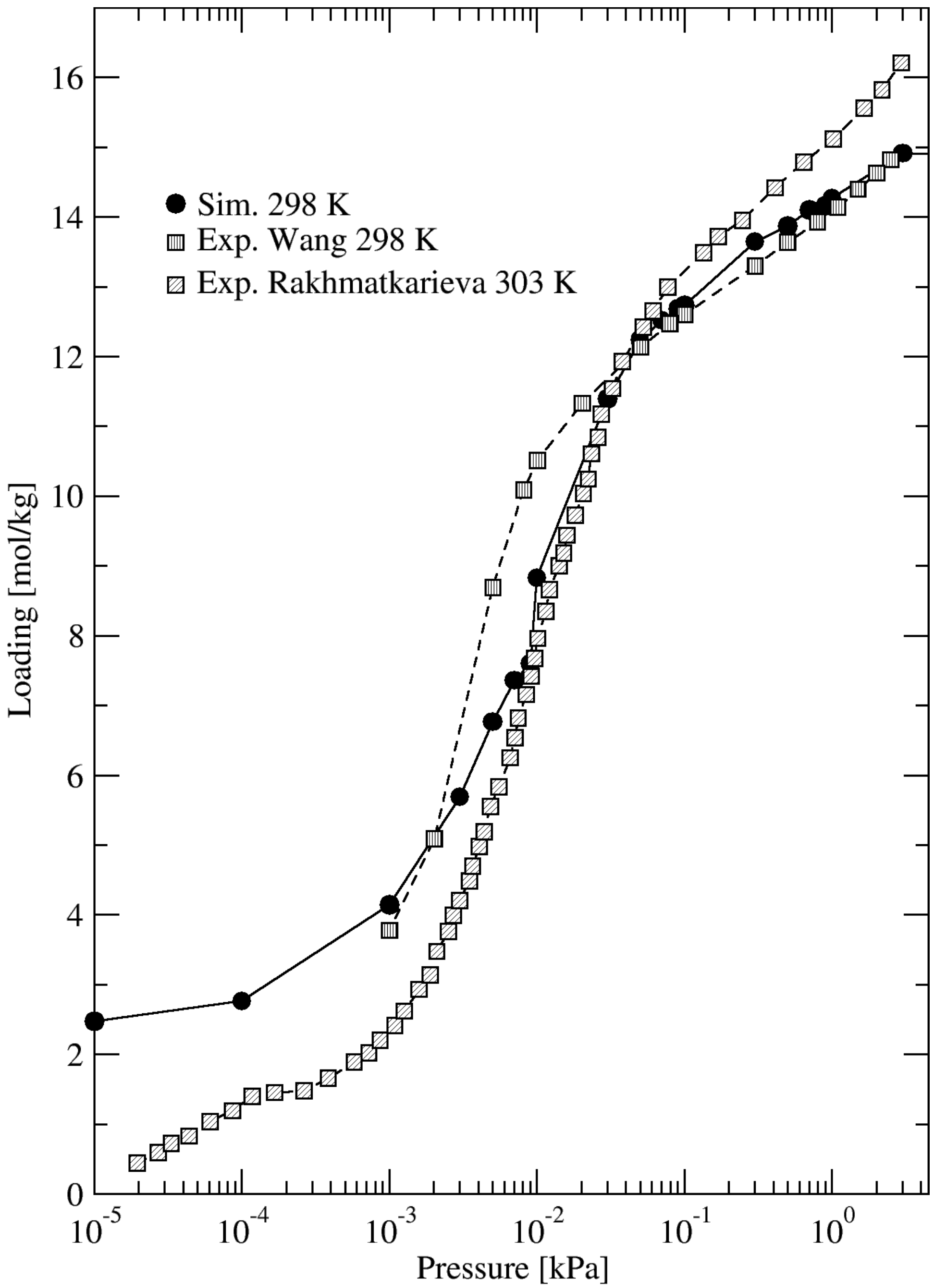

Figure S7. Simulated (circles) and experimental[1,2] (squares) adsorption isotherms of water in 4A zeolite.

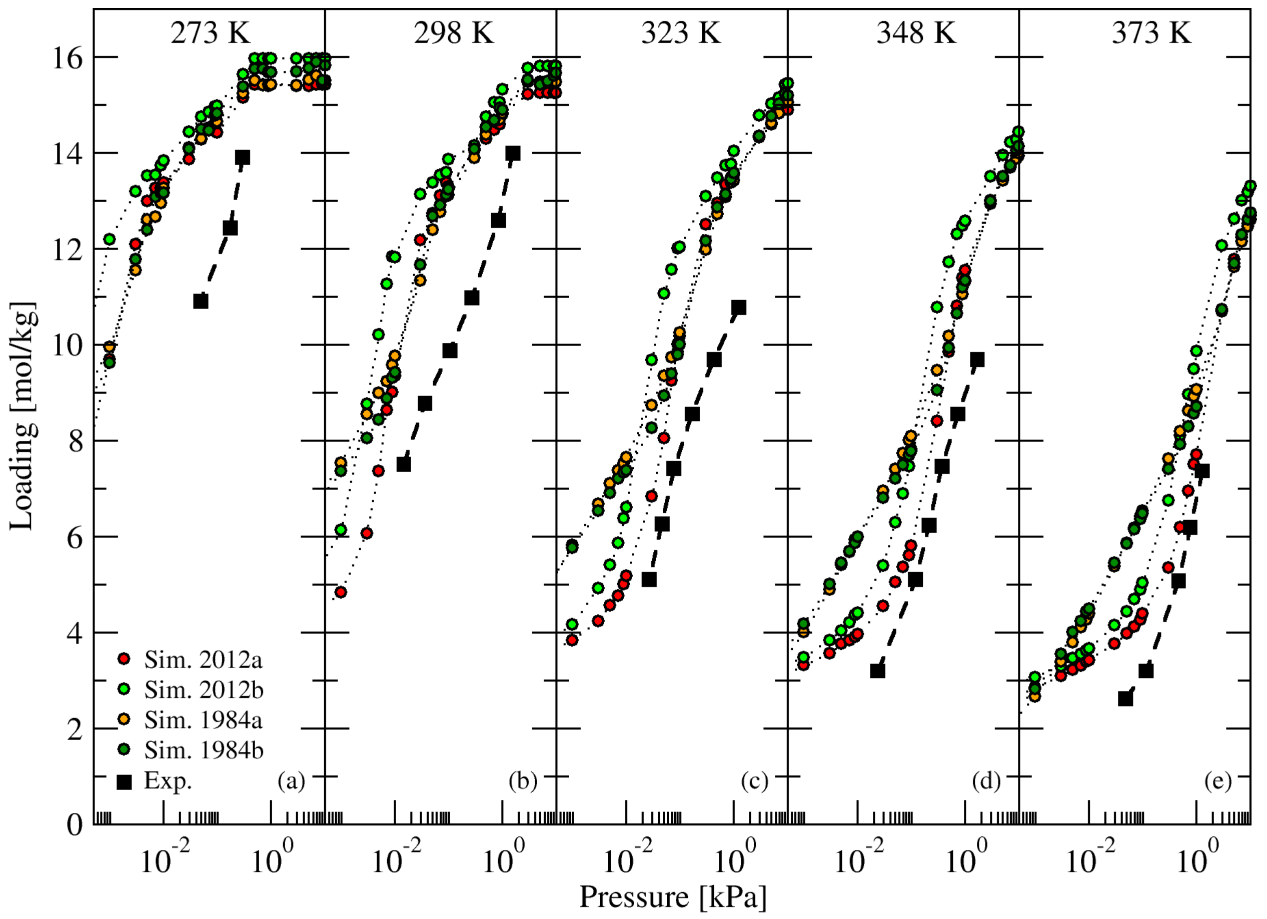

Figure S8. Water adsorption isotherms in 5A zeolite structures with 2Ca+8Na (2012a and 2012b) and 5Ca+2Na (1984a and 1984b) per cage[3-8].

References

(1) Rakhmatkarieva, F.; Davlatova, O.; Kokhkharov, M.; Xudoyberganov, M.; Ergashev, O.; Abdurakhmonov, E.; Abdulkhaev, T. Mechanism of H_2O Vapor Adsorption in A Type Zeolites: A Model Based on Adsorption Calorimetry. *E3S Web Conf.* **2023**, *434*, 03032. DOI: 10.1051/e3sconf/202343403032.

(2) Wang, Y. Measurements and Modeling of Water Adsorption Isotherms of Zeolite Linde-Type A Crystals. *Ind Eng Chem Res* **2020**, *59* (17), 8304-8314. DOI: 10.1021/acs.iecr.9b06891.

(3) Luhrs, H.; Derr, J.; Fischer, R. X. K and Ca exchange behavior of zeolite A. *Micropor Mesopor Mat* **2012**, *151*, 457-465. DOI: 10.1016/j.micromeso.2011.09.025.

(4) Broussard, L.; Shoemaker, D. P. The Structures of Synthetic Molecular Sieves. *J Am Chem Soc* **1960**, *82* (5), 1041-1051. DOI: 10.1021/ja01490a007.

(5) Seff, K.; Shoemaker, D. P. Structures of Zeolite Sorption Complexes .I. Structures of Dehydrated Zeolite 5A and Its Iodine Sorption Complex. *Acta Crystallogr* **1967**, *22*, 162-170. DOI: 10.1107/S0365110x67000283.

(6) Siegel, H.; Schollner, R.; Staudte, B.; Vandun, J. J.; Mortier, W. J. X-Ray Structural Investigations on Hydrothermally Treated (Ca_4, Na_4)-A Zeolites. *Zeolites* **1987**, *7* (4), 372-378. DOI: 10.1016/0144-2449(87)90042-X.

(7) Jang, S. B.; Song, S. H.; Kim, Y. Crystal structures of bromine sorption complexes of Ca^{2+}-exchanged zeolite A. *B Kor Chem Soc* **1995**, *16* (12), 1163-1167. DOI: 10.5012/bkcs.1995.16.12.1163.

(8) Adams, J. M.; Haselden, D. A. The Structure of Dehydrated Zeolite 5A (Si/Al = 1.02) by Neutron Profile Refinement. *J Solid State Chem* **1984**, *51* (1), 83-90. DOI: 10.1016/0022-4596(84)90318-9.
